# Thermophysical Properties of Alkanone + Aromatic Amine Mixtures at Varying Temperatures

**DOI:** 10.3389/fchem.2022.868836

**Published:** 2022-05-26

**Authors:** Aditi Prabhune, Amrita Natekar, Ranjan Dey

**Affiliations:** ^1^ Department of Chemistry, BITS-Pilani K. K. Birla Goa Campus, Zuarinagar, India; ^2^ Department of Chemistry, Dnyanprassarak Mandal’s College and Research Centre, Assagao-Bardez, India

**Keywords:** internal pressure, cohesive energy density (CED), excess entropy, isothermal compressibility, binary

## Abstract

In the present investigation, an attempt has been made to evaluate internal pressure $\left(P_{i}\right)$ , energy $\left(\Delta E_{vap}\right)$ , and enthalpy of vaporization $\left(\Delta H_{vap}\right)$ along with excess entropy $\left(S^{E}\right)\;$ and excess isothermal compressibility $\left(\beta_{T}^{E}\right)$ for binary solutions of alkanones (2-propanone, 2-butanone, and 2-heptanone) and aromatic amines (aniline, N-methylaniline, and pyridine) at 293.15, 298.15, and 303.15 K, respectively. The cohesive energy density (CED) and solubility parameter (δ) are studied to understand the strength of molecular interactions. The coefficient of thermal expansion (α) and isothermal compressibility $\left(\beta_{T}\right)$ have also been investigated using empirical equations and have been employed to understand the molecular interactions. All the evaluated properties have been used to understand the nature and extent of intermolecular interactions taking place. The observed trends in the properties and their variations have been discussed in terms of varying chain lengths of the alkyl group and the hydrogen bonding capability of the components. The findings show that the extent of interactions follows an order: aniline > NMA > pyridine, keeping the alkanone constant at all the temperatures under study.

## Introduction

Volumetric, acoustic, and thermophysical properties of nonaqueous binary mixtures provide valuable information about molecular interactions in systems resulting from solute–solute, solvent–solvent, solute–solvent interactions, structural effects, molecular orientation, energy changes, and free volume (Hemmat et al., 2017; Shakila et al., 2020; Jóźwiak et al., 2021). Knowledge of these properties has considerable significance in theoretical and applied areas of research (Rehman et al., 2020; Ezazi et al., 2021; Li et al., 2021; Nain, 2021; Sharma et al., 2021; Wan et al., 2021). Over the past several decades, internal pressure has played a key role in the study of the thermodynamics of liquid mixtures as it provides insights into the internal structure, clustering, structure making, and breaking along with various intermolecular interactions, namely, ionic, dipole–dipole interaction, and dipole-induced dipole attraction. (Marcus, 2013).

Two more significant thermophysical parameters, namely, the energy of vaporization $\left(\Delta E_{vap}\right)$ and enthalpy of vaporization $\left(\Delta H_{vap}\right)$ , have also been studied in the present investigation. Internal pressure can be used to evaluate the energy of vaporization (Pandey et al., 2020). Energy and enthalpy of vaporization coupled with the entropy of liquid mixtures provide an in-depth understanding and knowledge about the behavior of the system. Further insights and a deeper understanding of molecular interactions are provided by the knowledge of the corresponding excess parameters, that is, $\;\beta_{T}^{E},\;\;S^{E}$ (Hildebrand, 1947; Nain, 2008).

Alkanone and amine mixtures help us to gain insights into interactions in the amide solution due to the presence of carbonyl and amine groups in the component molecules. It is a well-known fact that proteins and amino acids are linked to each other by peptide bonds. For a better knowledge of biologically complex molecules, the first step involves understanding the intermolecular interactions in the liquid mixtures involving the amide functional group (Alonso et al., 2010a). Aniline is used in the manufacture of polyurethanes and in the pharmaceutical industry to produce drugs such as paracetamol. Aniline is also used in the synthesis of dyes, rubber, etc. N-Methylaniline (NMA) is used as an intermediate for dyes, agrochemicals, and other organic products. It is also used as a coupling solvent. Pyridine, an important raw material of chemical industries, is used as a precursor for the synthesis of various organic products in pharmaceutical and agrochemical industries (Alonso et al., 2011a; Kijevčanin et al., 2013). The increase in atmospheric CO_2_ due to the increase in fossil fuel combustion is one of the factors affecting global climate change. Newly developed modes of CO_2_ capture can help reduce atmospheric CO_2_ concentration significantly over traditional modes. Solvents and solid sorbents such as supported amine, ammonium material, and metal–organic frameworks (MOFs) are widely used for the process (Sanz -Pérez et al., 2016). The combination of aqueous alkanolamine solution with ionic liquid has reported anticorrosion protection property and acts as a better CO_2_ capture solvent (Bernard et al., 2016; Varghese and Karanikolos, 2020). Also, alkanolamine and room temperature ionic liquid emulsions are practicable to capture CO_2_ through crystallization of CO_2_-captured products (Hasib-ur-Rahman et al., 2012). The carbonated aqueous mixtures of alkanolamines and ionic liquids are also studied to understand the carbon steel corrosion behavior (Ali et al., 2012). A comprehensive review of the literature reveals that there is scarcity, pertaining to the thermophysical properties of these industrially significant binary mixtures, which has prompted the present work.

In the present investigation, internal pressure $\left(P_{i}\right)$ , energy $\left(\Delta E_{vap}\right)$ , enthalpy of vaporization $\left(\Delta H_{vap}\right)$ , cohesive energy density (*CED*), solubility parameter (δ) , free volume $\left(V_{f}\right)$ , excess entropy $\left(S^{E}\right)$ , and excess isothermal compressibility $\left(\beta_{T}^{E}\right)$ have been evaluated at three different temperatures, that is, 293.15, 298.15, and 303.15 K, for alkanones and aromatic amine mixtures. The experimental data required for the evaluation of the aforementioned properties have been taken from the literature and listed in Supplementary Table S1 (Alonso et al., 2010a; Alonso et al., 2010b; Alonso et al., 2011a).

The investigated systems comprise binary mixtures of the following:

1. 2-Propanone + (aniline/N-methylaniline/pyridine).
2. 2-Butanone + (aniline/N-methylaniline/pyridine).
3. 2-Heptanone + (aniline/N-methylaniline/pyridine).

## Theory

The change in intermolecular interactions upon mixing of the liquids is directly affected by the change in the volume and internal energy of the liquid mixture. Internal pressure can be interpreted as the volume derivative of internal energy (dU) in a constant temperature process, that is, ${\left(dU/dV\right)}_{T}$ . Internal pressure $\left(P_{i}\right)$ , a well-defined thermodynamic property of pure liquids, mixtures, and solutions, is derived from the thermodynamic equation of state through an expression that employs isothermal compressibility $\left(\beta_{T}\right)$ and the coefficient of thermal expansion (α) , two very significant thermophysical parameters (Suryanarayana, 1986; Marcus, 2013).

Internal pressure $\left(P_{i}\right)$ (Marcus, 2013; Almasi, 2020a) has been evaluated with the help of the following equation:

$$P_{i}=\;\frac{\alpha T}{\beta_{T}}-P,$$ (1)

where α is the coefficient of thermal expansion, $\beta_{T}$ is the isothermal compressibility, and *P* is the atmospheric pressure. The high magnitude of the first term in the right-hand side of Eq 1 renders the value of ‘ P ’ to be considered negligible. The values of the coefficient of thermal expansion (α) and isothermal compressibility $\left(\beta_{T}\right)$ have been evaluated by the method given elsewhere (Alonso et al., 2010b).

The internal pressure consists of attractive and repulsive forces between molecules. It is observed that when internal pressure is plotted against volume for a typical liquid at high volume (low P, high T), the attractive forces dominate $P_{i}$ , and it can be represented by the attraction coefficient of the van der Waals equation only (Barton, 1975). Hence, the attractive part of the internal energy (ΔU) is equal to the energy of vaporization $\left(\Delta E_{vap}\right)$ at low gas pressure, that is, $\left(\Delta U\right)=\left(\Delta E_{vap}\right)$ (Barton, 1975; Rastogi and Misra, 1995).

The energy of vaporization $\left(\Delta E_{vap}\right)$ is the energy required to break all the forces associated with 1 mol of liquid during the removal of that mole from liquid to vapor state. The energy of vaporization $\left(\Delta E_{vap}\right)$ (Pandey et al., 2020) is obtained by the following expression:

$${\left(\Delta E_{vap}\right)}_{mix}=\;\left(\frac{\alpha_{mix}T}{\beta_{Tmix}}\right).V_{m}={\left(P_{i}\right)}_{mix}.V_{m},$$ (2)

where $V_{m}$ is the molar volume.

Enthalpy of vaporization $\left(\Delta H_{vap}\right)$ is the sum of internal energy and pressure–volume work performed by the system (Levine, 2011). The enthalpy of vaporization (Pandey et al., 2020) is given by the following equation:

$$\left(\Delta H_{vap}\right)=\left(\Delta E_{vap}\right)+RT,$$ (3)

where all the symbols have their usual meaning.

Excess entropy is calculated from free volume $\left(V_{f}\right)$ (Hildebrand, 1947; Nain, 2008) using the following equation:

$$S^{E}=R\left[lnV_{f}-\;\left(x_{1}lnV_{f,1}+x_{2}lnV_{f,2}\right)\right],$$ (4)

where $x_{1}$ and $x_{2}$ are mole fractions, and $\;V_{f,1}\;and\;\;V_{f,2,}$ are the free volumes of the constituent pure components that have been calculated by making use of the equation (Nain, 2008; Almasi, 2020a; Almasi, 2020b):

$$V_{f}=\frac{RT}{\left(P+P_{i}\right)}.$$ (5)

Since *P* is very small as compared to *P*_*i*_, it is neglected in Eq 5.

Free volume $\left(V_{f}\right)$ represents the existing free space between the molecules in the liquid, and it is a measure of cohesion and the degree of interaction in liquid mixtures (Rehman et al., 2020).

The cohesive energy density (CED) represents the total cohesion per volume of the liquid, and it occurs due to the intermolecular forces present within the liquid (Dack, 1975). The cohesive energy density (CED) (Dack, 1975; Almasi, 2020a; Marcus, 2020) has been evaluated using the energy of vaporization $\left(\Delta E_{vap}\right)$ and molar volume $\left(V_{m}\right)$ given by the following equation:

$$CED\;=\;\frac{\Delta E_{vap}}{V_{m}}.$$ (6)

The solubility parameter (δ) represents the strength of intermolecular interactions between solvent molecules (Weerachanchai et al., 2012; Marcus, 2020). It is given by Pandey et al. (2020):

$$\delta =\sqrt{CED}=\sqrt{\frac{\Delta E_{vap}}{V_{m}}}.$$ (7)

The coefficient of thermal expansion (α) and isothermal compressibility $\left(\beta_{T}\right)$ have been calculated using the following empirical equations (Shukla et al., 2011; Nanda et al., 2012):

$$\alpha =\frac{75.6\times 10^{-3}}{T^{1/9}u^{1/2}\rho^{1/3}},$$ (8)

$$\beta_{T}=\frac{17.1\times 10^{-3}}{T^{4/9}\rho^{4/3}u^{2}},$$ (9)

where u is the ultrasonic velocity, ρ is the density, and T is the temperature in Kelvin. The experimental data of ultrasonic velocity and density required for evaluation have been acquired from the literature (Alonso et al., 2010a; Alonso et al., 2010b; Alonso et al., 2011a).

## Results and Discussion

The binary systems comprising alkanones and aromatic amines have been studied at three different temperatures (293.15, 298.15, and 303.15 K). Internal pressure, energy, and enthalpy of vaporization together with excess entropy have been evaluated employing thermodynamic properties to understand the intermolecular interactions present in the systems under investigation. All the requisite thermophysical properties of the pure components have been taken from the literature (Alonso et al., 2010a; Alonso et al., 2010b; Alonso et al., 2011a).

The coefficient of thermal expansion (α) and isothermal compressibility $\left(\beta_{T}\right)$ are two critical thermodynamic properties to understand the nature and extent of the interactions taking place in the liquid mixtures. The evaluated values of both properties at 298.15 K are recorded in Supplementary Table S2 (Alonso et al., 2010a; Alonso et al., 2010b; Alonso et al., 2011a). The coefficient of thermal expansion (α) is defined as the relative change in the volume with the temperature under isobaric conditions, and it is expressed in the study by Rama Rao et al. (2021):

$$\alpha =\frac{1}{V}{\left(\frac{\partial V}{\partial T}\right)}_{P}.$$ (10)

The α values in the present investigation have been evaluated from the $\alpha^{E}$ values obtained from the literature (Alonso et al., 2010a; Alonso et al., 2010b; Alonso et al., 2011a). It is observed that the α values show an increasing trend with the increase in the concentration of the alkanones for all the systems. The variation in the $\alpha^{E}$ values, as seen from the literature (Alonso et al., 2010a; Alonso et al., 2010b; Alonso et al., 2011a), clearly indicates a higher degree of interactions existing between the unlike molecules.

Isothermal compressibility $\left(\beta_{T}\right)$ is the relative change in the volume with pressure under isothermal conditions. It is expressed in the study by Rama Rao et al., (2021):

$$\beta_{T}=-\frac{1}{V}{\left(\frac{\partial V}{\partial P}\right)}_{T}.$$ (11)

In the present investigation, isothermal compressibility $\left(\beta_{T}\right)$ has been evaluated using the following expression (Alonso et al., 2010a):

$$\beta_{T}=\;\beta_{s}+\frac{T\;V\;\alpha^{2}}{C_{p}}.$$ (12)

Excess isothermal compressibility has been evaluated using the following expression (Alonso et al., 2010a):

$$\beta_{T}^{E}=\beta_{T}-\beta_{T}^{id}\;\text{and}\;\beta_{T}^{id}={ф}_{1}\beta_{T,1}+{ф}_{2}\beta_{T,2},$$ (13)

where ${ф}_{i}$ is volume fraction, and $\left(\beta_{T,i}\right)$ is the isothermal compressibility of constituent components.

The required experimental data of isentropic compressibility $\left(\beta_{s}\right)$ , molar volume (V) , isobaric heat capacity $\left(C_{p}\right)$ , and thermal expansivity (α) for evaluation of $\beta_{T}$ are obtained from the literature (Alonso et al., 2010a; Alonso et al., 2010b; Alonso et al., 2011a). It is seen that $\beta_{T}$ values increase with the addition of alkanone, indicating that liquid mixtures have become more compressible (Supplementary Table S2). Both α and $\beta_{T}$ expressions relate to the volume of the liquid components. With liquids being an intermediate phase between solids and gases, the expansion and compression studies play a vital role in understanding the molecular behavior and structural effects in the liquids.

**FIGURE 1:**
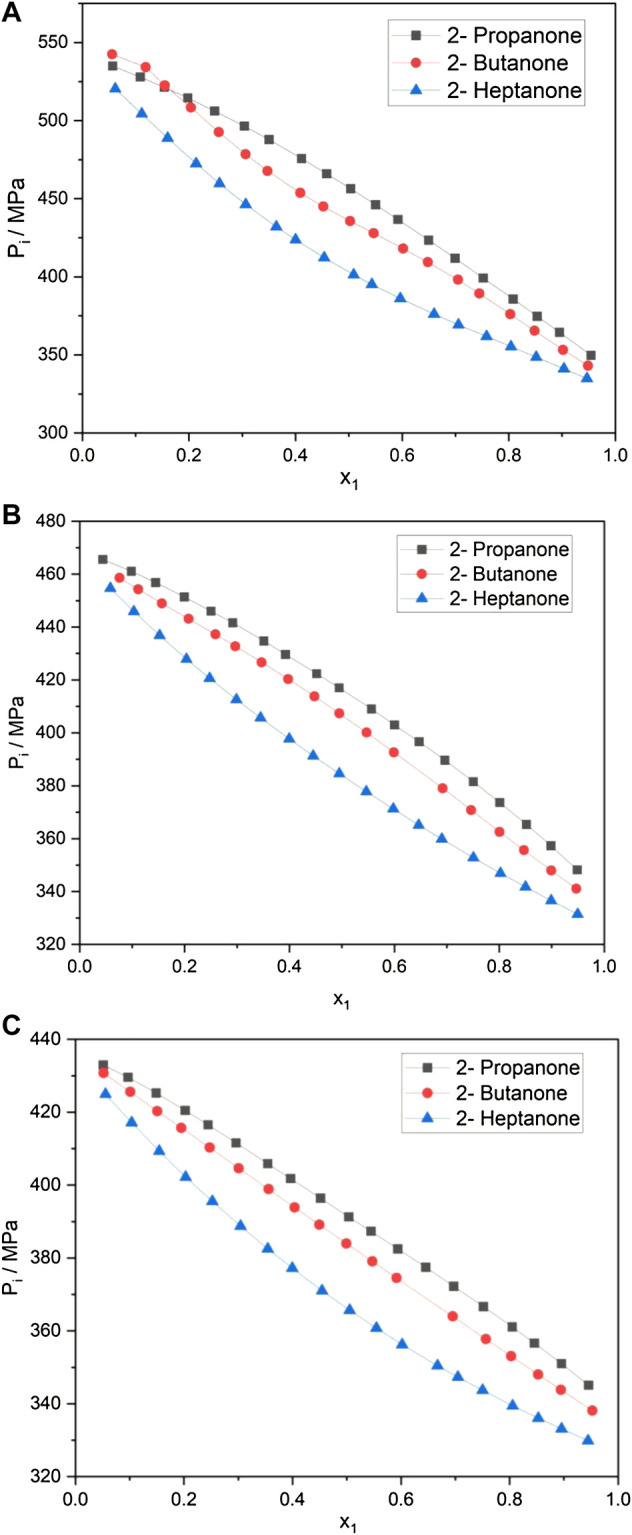
**(A)** Internal pressure of alkanone + aniline at 298.15 K. **(B)** Internal pressure of alkanone + N-methylaninline at 298.15 K. **(C)** Internal pressure of alkanone + pyridine at 298.15.

The internal pressure $\left(P_{i}\right)$ has been evaluated by Eq. 1 (Marcus, 2013; Almasi, 2020b; Rehman et al., 2020) using the knowledge of two significant parameters, namely, the coefficient of thermal expansion (α) and isothermal compressibility $\left(\beta_{T}\right)$ , and is presented in Table 1 for 298.15 K. The values of $P_{i}$ for all the systems under consideration tend to decrease with an increase in the mole fraction of the first component, that is, alkanone. The $P_{i}$ values are seen to range from 534.90 to 345.08 MPa for 2-propanone systems, 542.42 to 338.10 MPa for 2-butanone systems, and 520.42 to 329.87 MPa for 2-heptanone systems at 298.15 K. Figure 1A exhibits the variation in the internal pressure values for the alkanone + aniline systems at 298.15 K over the entire mole fraction range. A look at Figure 1A indicates a decreasing trend in the $P_{i}$ values for alkanone + aniline systems with an increase in the mole fraction of the alkanones. On average, the highest $P_{i}$ values are seen to be those of 2-propanone + aniline and the lowest are those of 2-heptanone + aniline, with the 2-butanone + aromatic amine mixtures giving intermediate values.

**TABLE 1:** Thermodynamic properties of alkanones + aromatic amines at 298.15 K (Alonso et al., 2010a; Alonso et al., 2010b; Alonso et al., 2011a).

$x_{1}$	$P_{i}$ /MPa	$\Delta E_{vap}$ /KJ-mol^−1^	$\Delta H_{vap}$ /KJ-mol^−1^	CED/J-mol^−1^cm^−3^	δ/(J-mol^−1^cm^−3^)^1/2^	$V_{f}$ /cm^3^-mol^−1^	$S^{E}$ /J-mol^−1^K^−1^
2-Propanone + aniline							
0.0569	534.90	48.33	50.80	533.81	23.10	4.63	0.1183
0.1088	527.91	47.13	49.61	525.87	22.93	4.70	0.2117
0.1539	521.42	46.06	48.54	518.56	22.77	4.75	0.2849
0.1978	514.54	45.00	47.47	510.97	22.60	4.82	0.3459
0.2484	506.16	43.74	46.22	501.81	22.40	4.90	0.4071
0.3039	496.46	42.35	44.83	491.39	22.17	4.99	0.4629
0.3505	487.81	41.17	43.64	482.19	21.96	5.08	0.4988
0.4114	475.70	39.59	42.06	469.60	21.67	5.21	0.5275
0.4585	465.98	38.37	40.84	459.60	21.44	5.32	0.5398
0.5035	456.38	37.20	39.67	449.86	21.21	5.43	0.5426
0.5501	446.12	35.98	38.46	439.56	20.97	5.56	0.5354
0.5922	436.73	34.90	37.37	430.17	20.74	5.68	0.5230
0.6499	423.39	33.41	35.89	417.09	20.42	5.85	0.4905
0.6993	411.91	32.17	34.64	405.95	20.15	6.02	0.4548
0.7522	399.23	30.85	33.32	393.88	19.85	6.21	0.4015
0.8082	385.75	29.48	31.95	381.19	19.52	6.43	0.3347
0.8533	374.67	28.39	30.87	370.97	19.26	6.62	0.2684
0.8951	364.43	27.41	29.89	361.62	19.02	6.80	0.2013
0.9545	349.72	26.05	28.52	348.41	18.67	7.09	0.0907
2-Propanone + N-methylaniline							
0.0440	465.54	50.03	52.51	465.12	21.57	5.32	0.0701
0.0984	461.07	48.61	51.09	460.15	21.45	5.38	0.1365
0.1447	456.83	47.38	49.86	455.52	21.34	5.43	0.1845
0.1996	451.39	45.90	48.38	449.63	21.20	5.49	0.2329
0.2503	446.01	44.53	47.00	443.89	21.07	5.56	0.2698
0.2919	441.62	43.41	45.89	439.17	20.96	5.61	0.2996
0.3511	434.71	41.79	44.27	431.92	20.78	5.70	0.3281
0.3924	429.67	40.67	43.15	426.73	20.66	5.77	0.3425
0.4520	422.36	39.08	41.56	419.19	20.47	5.87	0.3605
0.4948	417.01	37.94	40.42	413.70	20.34	5.94	0.3698
0.5563	409.00	36.33	38.80	405.63	20.14	6.06	0.3743
0.6004	403.01	35.17	37.65	399.64	19.99	6.15	0.3705
0.6473	396.65	33.96	36.44	393.32	19.83	6.25	0.3647
0.6962	389.64	32.71	35.19	386.48	19.66	6.36	0.3483
0.7506	381.52	31.32	33.80	378.64	19.46	6.50	0.3198
0.8009	373.63	30.05	32.52	371.13	19.26	6.63	0.2817
0.8518	365.36	28.76	31.24	363.29	19.06	6.78	0.2327
0.8986	357.31	27.58	30.06	355.78	18.86	6.94	0.1737
0.9488	348.20	26.31	28.79	347.34	18.64	7.12	0.0941
2-Propanone + pyridine							
0.0514	432.96	34.85	37.33	432.77	20.80	5.73	0.0460
0.0969	429.58	34.43	36.90	429.22	20.72	5.77	0.0766
0.1486	425.26	33.91	36.39	424.73	20.61	5.83	0.1014
0.2023	420.47	33.36	35.84	419.76	20.49	5.90	0.1204
0.2444	416.54	32.92	35.40	415.71	20.39	5.95	0.1310
0.2960	411.59	32.37	34.85	410.62	20.26	6.02	0.1402
0.3544	405.87	31.75	34.23	404.81	20.12	6.11	0.1469
0.3965	401.79	31.30	33.78	400.64	20.02	6.17	0.1516
0.4516	396.40	30.73	33.20	395.19	19.88	6.25	0.1552
0.5036	391.29	30.19	32.66	390.04	19.75	6.34	0.1568
0.5448	387.34	29.77	32.25	386.05	19.65	6.40	0.1593
0.5940	382.47	29.26	31.74	381.20	19.52	6.48	0.1576
0.6454	377.44	28.75	31.22	376.20	19.40	6.57	0.1559
0.6971	372.21	28.22	30.70	371.07	19.26	6.66	0.1488
0.7518	366.65	27.67	30.15	365.62	19.12	6.76	0.1387
0.8049	361.08	27.13	29.60	360.20	18.98	6.87	0.1232
0.8456	356.64	26.70	29.18	355.90	18.87	6.95	0.1063
0.8955	351.02	26.17	28.65	350.47	18.72	7.06	0.0791
0.9456	345.08	25.63	28.11	344.78	18.57	7.18	0.0427
2-Butanone + aniline							
0.0560	542.42	49.51	51.99	541.36	23.27	4.57	0.2609
0.1188	534.20	48.53	51.01	531.11	23.05	4.64	0.3848
0.1547	522.59	47.43	49.91	519.39	22.79	4.74	0.3456
0.2038	508.32	46.03	48.51	504.38	22.46	4.88	0.3117
0.2563	492.57	44.50	46.98	488.02	22.09	5.03	0.2597
0.3064	478.42	43.13	45.61	473.38	21.76	5.18	0.2175
0.3474	467.79	42.11	44.59	462.46	21.50	5.30	0.1946
0.4089	453.71	40.76	43.24	448.05	21.17	5.46	0.1864
0.4523	444.97	39.92	42.40	439.14	20.96	5.57	0.1980
0.5026	435.66	39.04	41.52	429.75	20.73	5.69	0.2233
0.5467	427.91	38.31	40.79	421.98	20.54	5.79	0.2501
0.6019	418.05	37.40	39.87	412.28	20.30	5.93	0.2770
0.6481	409.30	36.60	39.08	403.79	20.09	6.06	0.2857
0.7048	398.10	35.59	38.07	392.99	19.82	6.23	0.2815
0.7451	389.22	34.81	37.29	384.63	19.61	6.37	0.2551
0.8030	376.06	33.66	36.14	372.26	19.29	6.59	0.2004
0.8487	365.42	32.74	35.22	362.31	19.03	6.78	0.1444
0.9019	353.22	31.70	34.18	351.05	18.74	7.02	0.0746
0.9489	343.02	30.84	33.32	341.85	18.49	7.23	0.0190
2-Butanone + N-methylaniline							
0.0760	458.60	49.29	51.77	457.84	21.40	5.41	0.0195
0.1115	454.28	48.49	50.97	453.25	21.29	5.46	0.0415
0.1566	448.94	47.51	49.99	447.59	21.16	5.52	0.0709
0.2076	443.12	46.42	48.90	441.40	21.01	5.59	0.1068
0.2589	437.20	45.35	47.82	435.18	20.86	5.67	0.1405
0.2967	432.72	44.55	47.03	430.51	20.75	5.73	0.1618
0.3471	426.67	43.49	45.97	424.22	20.60	5.81	0.1876
0.3973	420.35	42.43	44.91	417.73	20.44	5.90	0.2057
0.4477	413.83	41.36	43.84	411.08	20.28	5.99	0.2185
0.4950	407.35	40.35	42.83	404.58	20.11	6.09	0.2214
0.5471	400.09	39.23	41.71	397.31	19.93	6.20	0.2194
0.5992	392.63	38.11	40.59	389.89	19.75	6.31	0.2106
0.6921	379.03	36.14	38.62	376.48	19.40	6.54	0.1806
0.7463	370.80	35.00	37.48	368.54	19.20	6.68	0.1518
0.8007	362.58	33.88	36.35	360.64	18.99	6.84	0.1194
0.8470	355.63	32.95	35.42	354.05	18.82	6.97	0.0898
0.8993	347.93	31.92	34.40	346.78	18.62	7.12	0.0560
0.9472	341.06	31.03	33.51	340.38	18.45	7.27	0.0259
2-Butanone + pyridine							
0.0517	430.72	35.02	37.50	430.50	20.75	5.76	-0.0141
0.1009	425.57	34.78	37.26	425.17	20.62	5.82	-0.0035
0.1510	420.32	34.53	37.01	419.75	20.49	5.90	0.0057
0.1952	415.70	34.31	36.79	414.99	20.37	5.96	0.0132
0.2473	410.28	34.05	36.53	409.41	20.23	6.04	0.0209
0.3010	404.63	33.77	36.25	403.67	20.09	6.13	0.0264
0.3559	398.88	33.49	35.97	397.83	19.95	6.21	0.0307
0.4041	393.85	33.24	35.71	392.76	19.82	6.29	0.0334
0.4494	389.15	33.00	35.48	388.01	19.70	6.37	0.0353
0.4994	383.99	32.73	35.21	382.84	19.57	6.46	0.0366
0.5472	379.07	32.48	34.96	377.91	19.44	6.54	0.0367
0.5916	374.50	32.25	34.72	373.38	19.32	6.62	0.0358
0.6951	363.97	31.70	34.17	362.97	19.05	6.81	0.0311
0.7563	357.77	31.37	33.85	356.91	18.89	6.93	0.0257
0.8029	353.03	31.12	33.59	352.30	18.77	7.02	0.0196
0.8525	348.04	30.85	33.33	347.45	18.64	7.12	0.0125
0.8944	343.85	30.62	33.10	343.41	18.53	7.21	0.0060
0.9525	338.10	30.31	32.79	337.88	18.38	7.33	−0.0039
2-Heptanone + aniline							
0.0619	520.42	49.13	51.61	519.50	22.79	4.76	−0.0477
0.1115	504.35	48.78	51.26	502.77	22.42	4.91	−0.1005
0.1601	488.77	48.39	50.86	486.74	22.06	5.07	−0.1578
0.2133	472.46	47.96	50.44	470.05	21.68	5.25	−0.2168
0.2575	459.73	47.63	50.10	457.04	21.38	5.39	−0.2588
0.3064	446.36	47.28	49.76	443.53	21.06	5.55	−0.2991
0.3638	432.00	46.95	49.43	429.03	20.71	5.74	−0.3304
0.4002	423.69	46.78	49.26	420.59	20.51	5.85	−0.3394
0.4537	412.18	46.58	49.06	409.10	20.23	6.01	−0.3442
0.5092	401.34	46.44	48.92	398.32	19.96	6.18	−0.3331
0.5430	395.19	46.39	48.86	392.24	19.81	6.27	−0.3198
0.5964	386.10	46.34	48.82	383.33	19.58	6.42	−0.2895
0.6599	376.11	46.33	48.81	373.59	19.33	6.59	−0.2412
0.7057	369.32	46.34	48.82	367.02	19.16	6.71	−0.2008
0.7584	361.76	46.36	48.84	359.78	18.97	6.85	-−0.1520
0.8041	355.30	46.36	48.84	353.61	18.80	6.98	−0.1101
0.8515	348.61	46.34	48.82	347.26	18.63	7.11	−0.0696
0.9033	341.20	46.27	48.75	340.26	18.45	7.27	−0.0311
0.9467	334.84	46.17	48.65	334.25	18.28	7.40	−0.0057
2-Heptanone + N-methylaniline							
0.0583	454.70	50.39	52.87	454.17	21.31	5.45	−0.0520
0.1032	445.87	50.01	52.49	445.08	21.10	5.56	−0.0809
0.1524	436.79	49.65	52.13	435.76	20.87	5.68	−0.1049
0.2036	427.86	49.30	51.78	426.65	20.66	5.79	−0.1236
0.2475	420.65	49.03	51.51	419.29	20.48	5.89	−0.1338
0.2987	412.55	48.74	51.22	411.09	20.28	6.01	−0.1424
0.3448	405.65	48.50	50.98	404.09	20.10	6.11	−0.1450
0.3994	397.71	48.23	50.71	396.12	19.90	6.23	−0.1461
0.4452	391.31	48.01	50.49	389.69	19.74	6.33	−0.1442
0.4949	384.59	47.78	50.26	382.97	19.57	6.45	−0.1398
0.5465	377.75	47.55	50.03	376.19	19.40	6.56	−0.1347
0.5977	371.24	47.34	49.82	369.76	19.23	6.68	-0.1263
0.6464	365.15	47.14	49.61	363.79	19.07	6.79	−0.1182
0.6904	359.82	46.96	49.44	358.59	18.94	6.89	−0.1091
0.7506	352.81	46.73	49.21	351.74	18.75	7.03	−0.0928
0.8023	346.93	46.55	49.02	346.06	18.60	7.14	−0.0780
0.8499	341.73	46.38	48.86	341.05	18.47	7.25	−0.0613
0.8989	336.56	46.23	48.71	336.09	18.33	7.37	−0.0418
0.9494	331.43	46.09	48.57	331.19	18.20	7.48	−0.0185
2-Heptanone + pyridine							
0.0553	424.96	35.77	38.25	424.90	20.61	5.83	−0.0613
0.1034	417.10	36.30	38.78	416.98	20.42	5.94	−0.1014
0.1543	409.31	36.87	39.35	409.15	20.23	6.06	−0.1362
0.2032	402.21	37.40	39.88	402.02	20.05	6.16	−0.1647
0.2521	395.52	37.94	40.41	395.31	19.88	6.27	−0.1873
0.3042	388.72	38.49	40.97	388.49	19.71	6.38	−0.2068
0.3542	382.51	39.02	41.50	382.29	19.55	6.48	−0.2209
0.3994	377.19	39.50	41.98	376.96	19.42	6.57	−0.2293
0.4544	371.04	40.08	42.56	370.81	19.26	6.68	−0.2343
0.5053	365.64	40.61	43.09	365.42	19.12	6.78	−0.2343
0.5544	360.74	41.13	43.61	360.54	18.99	6.87	−0.2291
0.6019	356.25	41.63	44.11	356.06	18.87	6.96	−0.2196
0.6671	350.47	42.32	44.80	350.31	18.72	7.07	−0.1996
0.7047	347.30	42.73	45.20	347.16	18.63	7.14	−0.1851
0.7500	343.69	43.22	45.69	343.57	18.54	7.21	−0.1636
0.8056	339.47	43.82	46.30	339.38	18.42	7.30	−0.1332
0.8528	336.06	44.33	46.81	335.99	18.33	7.38	−0.1043
0.8956	333.11	44.80	47.28	333.07	18.25	7.44	−0.0751
0.9444	329.87	45.33	47.81	329.86	18.16	7.51	−0.0395

Figures 1B, C show a similar trend in the $P_{i}$ values for both alkanone + N-methylaniline (NMA) and alkanone + pyridine at 298.15 K with the increase in the mole fraction of the corresponding alkanone. Figure 1C shows that the initial values of $P_{i}$ for alkanone + pyridine are lower than those of the other alkanone + aromatic amine systems. The $P_{i}$ values for all the systems with pyridine show relatively less variation with values tightly bunched together.

The internal pressure of a liquid accounts for the change in internal energy with volume under isothermal conditions and arises due to the presence of various forces such as repulsion, dispersion, and ionic and dipole interaction, which contribute to the overall cohesion in the liquid system. The decrease in $P_{i}$ for all systems indicates the presence of strong adhesive forces (Saini et al., 2021; Baluja et al., 2010). The variation in $P_{i}$ values of alkanone with aniline and NMA points toward specific interactions taking place with strong adhesion. The smaller variation in the $P_{i}$ values of alkanone with pyridine indicate relatively less interactions taking place. An overview of Figures 1A–C reveals that the extent of interactions follows an order: aniline > NMA > pyridine, keeping the alkanone constant at 298.15 K. The trends observed in $P_{i}$ values of alkanone + aromatic amine systems at 298.15 K and their values at equimolar concentration are in good agreement with the literature data (Alonso et al., 2011b).

**FIGURE 2:**
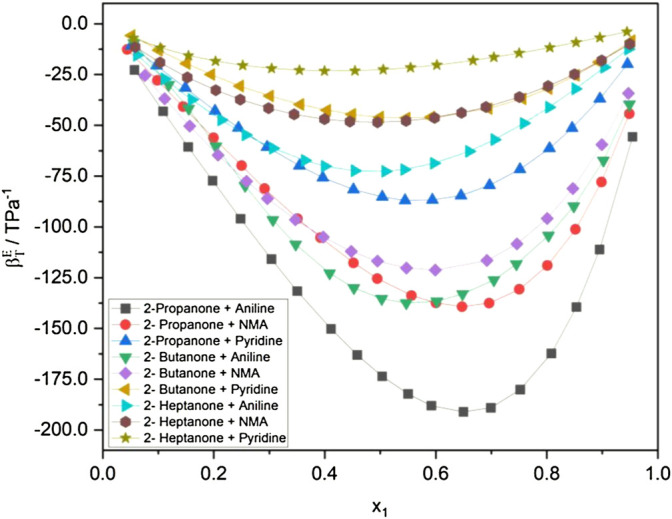
Excess isothermal compressibility at 298.15 K.

A plot of excess isothermal compressibility $\left(\beta_{s}^{E}\right)$ in Figure 2 encompasses all the systems, and these values are found to be negative over the entire composition range (Nain, 2013). The highest negative values of excess isothermal compressibility are observed between 0.5000 and 0.7000 mol fraction range for all the alkanone + aromatic amine mixtures at 298.15 K, indicating the presence of strong interactions in this composition range. The highest negative value of $\beta_{T}^{E}$ is observed to be that of 2-propanone + aniline and the least for 2-heptanone + pyridine with all other systems lying between them. It is observed that alkanone + pyridine systems show lower values of *P*_*i*_ than alkanone + aniline and alkanone + NMA systems. Figure 2 implies that the molecular interactions occurring in the liquid mixture follow the order: aniline > NMA > pyridine for a particular set of alkanones at 298.15 K. The higher negative trend in the values of excess isothermal compressibility points toward a higher extent of the interactions between the unlike molecules resulting from the good geometrical fitting of the constituent components. The excess molar volume $\left(V_{m}^{E}\right)$ and excess isentropic compressibility $\left(\beta_{s}^{E}\right)$ values reported in the literature are found to be showing similar trends and validate the observations (Alonso et al., 2010a; Alonso et al., 2010b; Alonso et al., 2011a).

The values of energy of vaporization $\left(\Delta E_{vap}\right)$ recorded in Table 1 are found to decrease with the increment of the alkanone concentration for all the systems except for 2-heptanone + pyridine, where the values are seen to increase. The decrease in $\left(\Delta E_{vap}\right)$ values for alkanone + pyridine systems is seen to be lower than that of the other alkanone + aromatic amine mixtures at 298.15 K. The enthalpy of vaporization $\left(\Delta H_{vap}\right)$ shows similar trends for all alkanone + aromatic amine systems over the entire mole fraction range. The decreasing trend in both the thermodynamic properties suggests a decrease in the cohesive forces with the addition of the first component. The exception observed in 2-heptanone + pyridine may be resulting from relatively fewer interactions arising due to the presence of dominating cohesive forces.

The cohesive energy density (CED) and solubility parameter (δ) are evaluated using Eqs. 8, 9 (Almasi, 2020a; Almasi, 2020b) at 298.15 K and are recorded in Table 1. The cohesive energy density represents the total cohesion per volume of the liquid. Cohesion in a liquid is a resultant of the intermolecular forces, especially attractive forces evolving from hydrogen bonding and dipole–dipole and dispersion interactions. The cohesion creates around 1,000–10,000 atm pressure within the liquid. A solute molecule experiences this pressure when dissolved in the solvent, which increases as the interaction between solute–solvent molecules increases. This implies that the solution exists under higher internal pressure than the pure solvent. Even though $P_{i}$ and CED values are almost similar, they do not reflect the same physical property of the liquid. The internal pressure is a measure of nonspecific interaction energy within the liquid while the CED measures the total intermolecular interaction energy, which includes both specific and nonspecific interaction energies within the liquid mixture (Dack, 1975). The CED values (Table 1) lie between 541.36 J-mol^−1^-cm^−3^ and 348.41 J-mol^−1^-cm^−3^ and tend to decrease with the addition of alkanone for all systems at 298.15 K. The decrease in the CED indicates a reduction in the cohesive forces present in the liquid mixtures (Suryanarayana, 1986; Levine, 2011). The solubility parameter developed by Hildebrand and Scott (1950) and Dack (1975) is a square root value of CED. It indicates the strength of intermolecular interactions between solvent molecules (Weerachanchai et al., 2012). Both solvent and solute molecules must overcome the cohesion present in the liquid in order to dissolve into the liquid. The solubility of components is possible when interactive forces between components or cohesive energy values of the components are similar (Welker, 2012). The solubility parameter values presented in Table 1 show a decrease in the δ values with an increase in the alkanone component. Alkanone + aniline systems show relatively higher solubility parameter values, followed by alkanone + NMA and alkanone + pyridine systems at 298.15 K.

Free volume $\left(V_{f}\right)$ represents the existing free space between the molecules in the liquid, and it depends on the internal pressure of the liquid. It is a measure of cohesion and degree of interaction in liquid mixtures. The presence of attractive forces between the solute and solvent molecules causes an increase in unoccupied space or volume in the liquid mixture (Rehman et al., 2020). Free volume $\left(V_{f}\right)$ is evaluated using Eq 5 (Nain, 2008; Almasi, 2020a; Almasi, 2020b), and the values are recorded in Table 1. A gradual increase in the $V_{f}$ values is noted with the increase in the alkanone concentration for all the alkanone + aromatic amine systems at 298.15 K. This signifies an increase in the molecular association causing less cohesion in the mixtures (Baluja et al., 2010; Saini et al., 2021). It can be seen from Table 1 that alkanone + aniline systems have relatively more variations in the values of the properties than NMA and pyridine systems at 298.15 K.

**FIGURE 3:**
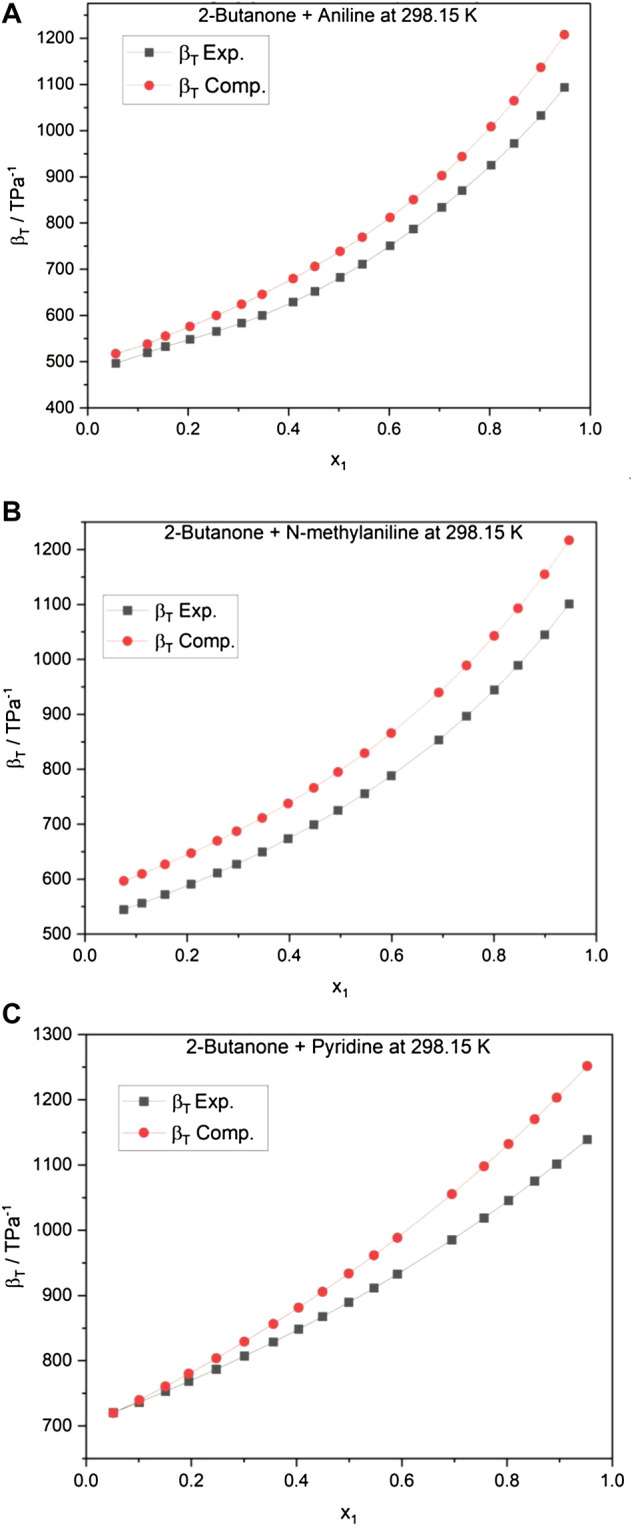
**(A)** Isothermal compressibility of 2-butanone + aniline at 298.15 K. **(B)** Isothermal compressibility of 2-butanone + N-methylaniline. **(C)** Isothermal compressibility of 2-butanone + pyridine at 298.15 K.

The thermal expansivity (α) and isothermal compressibility $\left(\beta_{T}\right)$ have also been evaluated using two well-known empirical equations (Eqs. 8, 9) (Shukla et al., 2011; Nanda et al., 2012). Both experimental and computed values are tabulated in Supplementary Table S2, and $\beta_{T}$ is graphically represented for 2-butanone + aromatic amine systems in Figures 3A–C. It can be seen from Figures 3A–C and Supplementary Table S2 that the computed values of α and $\beta_{T}$ are in good agreement with the experimental values at 298.15 K. The close agreement with the literature value at 298.15 K prompted the usage of the empirical equations (Eqs. 8, 9) at 293.15 and 303.15 K temperatures. These two parameters have then been utilized to compute internal pressure, energy and enthalpy of vaporization, free volume, cohesive energy density, solubility parameter, and excess entropy and are listed in Supplementary Tables S3, S4. A perusal of Supplementary Tables S3, S4 shows that all the alkanone + aromatic amine systems exhibit similar trends in these properties at 293.15 and 303.15 K as it has been observed at 298.15 K. A similar trend is observed at all three temperatures which follow the order: aniline > NMA > pyridine, for a similar set of alkanones.

Excess entropy $\left(S^{E}\right)$ (Hildebrand, 1947; Nain, 2008) evaluated at 298.15 K has been recorded in Table 1. A perusal of Table 1 shows that 2-propanone and 2-butanone systems have positive excess entropy values whereas the 2-heptanone systems have negative values. The highest value of $S^{E}$ for the 2-propanone + aniline system at 298.15 K is seen to be 0.5426 J/(mol-K) at the mole fraction (x_1_ = ) 0.5035 of the first component. At the same temperature, the lowest value of *S* ^*E*^ is −0.3440 J/(mol-K) when x_1_ is 0.4537 for 2-heptanone + aniline, whereas the excess entropy values for other systems lie in between them.

**FIGURE 4:**
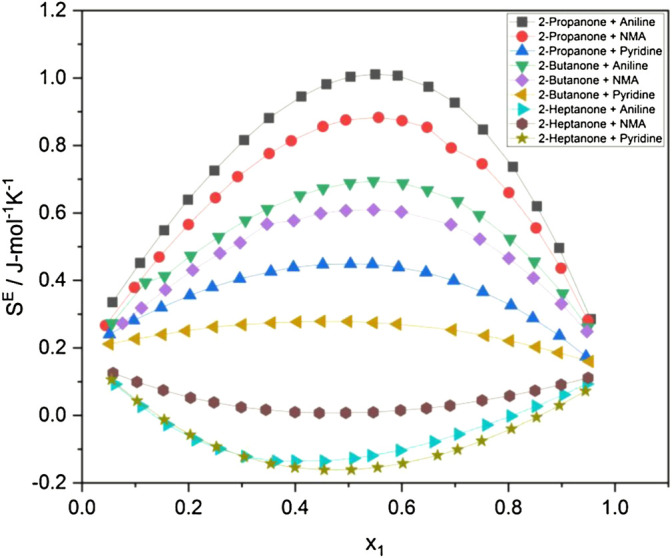
Excess entropy (comp.) at 298.15 K.

The excess entropy $\left(S^{E}\right)$ have also been evaluated by making use of the *P*_*i*_ values obtained through α and $\beta_{T}$ from the empirical equations at 298.15 K. Figure 4 depicts the computed $S^{E}$ values, and it is seen that trends similar to those of the experimental values of $S^{E}$ are observed at 298.15 K. The decrease in $S^{E}$ may be ascribed to weakening of the interaction between unlike molecules. The negative $S^{E}$ values of heptanone + aromatic amine systems show the least interactions between unlike molecules, which may have occurred due to structural effects.

The evaluated values of $V_{f}$ and *P*_*i*_ at the remaining two temperatures have been utilized to express $S^{E}$ at 293.15 K and 303.15 K and are represented graphically in Supplementary Figure S1, S2. The positive values of $S^{E}$ are observed for 2-propanone + aromatic amines and 2-butanone + aromatic amine systems while the 2-heptanone + aromatic amine systems show negative values at 293.15 and 303.15 K similar to the 298.15 K observations, as seen in Figure 4 and Table 1. With the increase in temperature, the excess entropy values tend to increase as the disorder is more in liquid systems at higher temperatures. In 2-heptanone systems, the negative values of excess entropy may be attributed to fewer interactions present in the systems.

**FIGURE 5:**
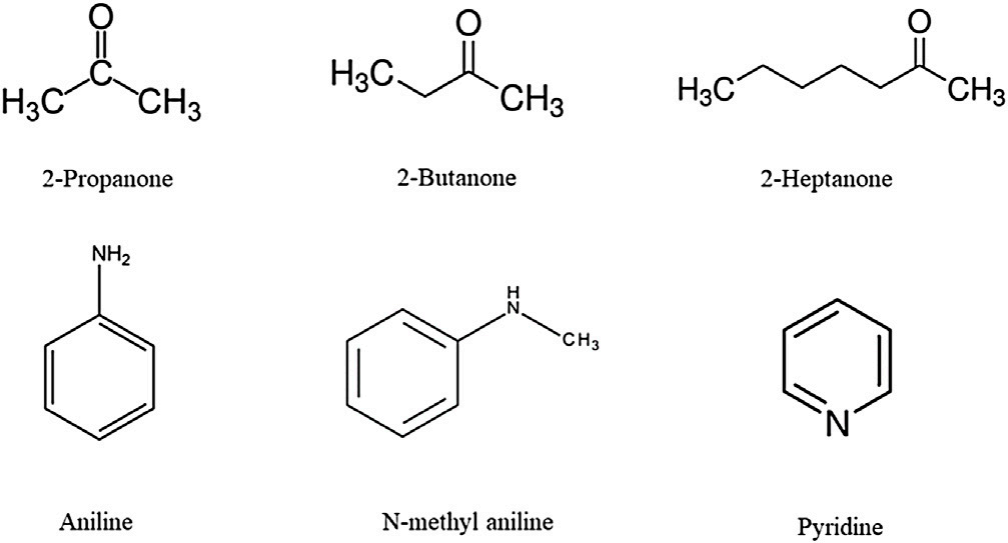
Chemical structures of components.

An overview of the plots in Figures 1A–C; Table 1; and Supplementary Tables S2, S3 reveals that while keeping alkanone constant, the extent of interactions follows the order: aniline > N-methylaniline > pyridine. These results are in excellent agreement with the $V_{m}^{E}$ values reported in the literature (Alonso et al., 2010a; Alonso et al., 2011a; Alonso et al., 2010b). The aforementioned order of interactions can also be attributed to the fact that alkanone + aniline interactions occur more readily than alkanone + N-methylaniline or alkanone + pyridine interactions due to the ability of aniline to form a hydrogen bond easily (Alonso et al., 2010a) as it is a primary amine whereas N-methylaniline and pyridine are secondary and tertiary amines, respectively. The chemical structures of constituent components are represented in Figure 5. Pyridine is freely soluble in water whereas aniline and NMA are slightly soluble in water (Kim et al., 2016). The solubility of alkanones in the aqueous phase is observed to be high for 2-propanone, followed by 2-butanone and then 2-heptanone (Kim et al., 2016).

The strong ability of aniline to form a hydrogen bond arises due to the presence of strong dipolar interactions among the aniline molecules (González et al., 2005) The presence of strong interactions between polar molecules is also observed by miscibility gaps between the upper critical solution temperatures (UCSTs) in liquid–liquid equilibria curves (Alonso et al., 2010b). For systems such as aniline + hexane and aniline + heptane, UCSTs are 342.7 and 343.11 K, respectively, while for pyridine systems, UCSTs with hexane and heptane are 252.1 and 255.2 K, respectively, which indicates the ability of aniline to have strong interactions with corresponding molecules (Alonso et al., 2010b). Aniline and NMA are self-associated liquids *via* hydrogen bonds in the pure state, and this self-association decreases when it is mixed with other alkanones (Alonso et al., 2010b). The structural effects are understood by the contribution to H^E^ for a system that includes positive values due to the breaking of propanone–propanone and aniline–aniline interactions while negative values are due to propanone–aniline interactions (Alonso et al., 2010b). The higher negative $H_{m}^{E}$ values of alkanone + aniline suggest that more negative contribution is added due to the interactions between unlike molecules compared to alkanone + NMA as the amine group in NMA is more sterically hindered (Alonso et al., 2011c).

The variation in molecular interactions when alkanone is varied and aromatic amines being kept constant follows the order: 2-propanone > 2-butanone > 2-heptanone. This may be attributed to the fact that as the chain length increases, the ability of amines to form a hydrogen bond with the oxygen atom of alkanone decreases. The C=O group in 2-propanone is more polar than that in 2-butanone or 2-heptanone, which makes it easier for 2-propanone to form a hydrogen bond with aniline. This fact is also supported by the dipole moment of the alkanones wherein 2-propanone has the highest dipole moment (2.88D) and 2-heptanone has the least (2.59 D) (Haynes, 2013). It is pertinent to point out that the steric restriction to the approaching alkanone molecule would be very high when the H atoms of the amino group in aniline are substituted by the methyl group (Nakanishi and Touhara, 1986).

It is evidenced from Figures 1A–C that the variation in the internal pressure values for an amine with the common alkanone exhibits a decreasing trend with the increase in temperature. The energy and enthalpy of vaporization values listed in Table 1 and Supplementary Tables S3, S4 show an increase with the change in temperature for an amine with a set of alkanones. It is observed that CED and solubility parameter values tend to decrease with an increase in temperature. Figure 4 and Supplementary Figures S1, S2 show that excess entropy values are increasing with the rise in temperature, indicating an increase in a disorder with the temperature in the liquid system.

## Conclusion

In the present investigation, internal pressure $\left(P_{i}\right)$ , energies and enthalpies of vaporization $\left(\Delta E_{vap}\;and\;\Delta H_{vap}\right)$ , cohesive energy density (CED), solubility parameter (δ), excess isothermal compressibility $\left(\beta_{T}^{E}\right)$ , and excess entropy $\left(S^{E}\right)$ have been evaluated. Due to the strong ability of aniline to form a hydrogen bond with the C=O group of alkanones, the systems containing aniline show maximum interactions. The highest interactions among all the systems are shown by 2-propanone + aniline, and this may be attributed to the higher polarity of 2-propanone. The presence of nitrogen in the pyridine ring lowers the tendency of forming hydrogen bonds; therefore, it results in systems with pyridine showing the least intermolecular interaction. Therefore, it can be concluded that thermophysical parameters coupled with excess properties can be used as a powerful tool for predicting the extent and nature of molecular interaction in the systems.

## Data Availability

The original contributions presented in the study are included in the article/Supplementary Material, further inquiries can be directed to the corresponding author.
