# Supplementary material for: Thermophysical Properties of Alkanone + Aromatic Amine Mixtures at Varying Temperatures
Source: Front Chem. 2022 May 26;10:868836. doi: 10.3389/fchem.2022.868836 (PMC9204321; doi:10.3389/fchem.2022.868836)

THERMOPHYSICAL PROPERTIES OF ALKANONE + AROMATIC AMINE MIXTURES AT VARYING TEMPERATURES

Aditi Prabhune ^a^, Amrita Natekar ^a,b^, Ranjan Dey ^a,^*

^a^ Department of Chemistry, BITS-Pilani K K Birla Goa Campus,

Zuarinagar, Goa- 403726, India.

^b^ Department of Chemistry, Dnyanprassarak Mandal’s College and Research Centre,

Assagao - Bardez, Goa- 403507, India.

*Email ID: [ranjandey@goa.bits-pilani.ac.in](mailto:ranjandey@goa.bits-pilani.ac.in)

SUPPLEMENATARY

**Table S1: Thermophysical properties of pure components at 298.15K [14,16,22].**

|  | *ρ /*  $g.\mathrm{cm}^{-3}$ | $u$*/*  $m.s^{-1}$ | $\alpha$ */*  ${10}^{-3}K^{-1}$ | $\beta_{s}$*/*  $\mathrm{TPa}^{-1}$ | $\beta_{T}$*/*  $\mathrm{TPa}^{-1}$ | $C_{p}$*/*  $J.\mathrm{mol}^{-1}.K^{-1}$ |
| --- | --- | --- | --- | --- | --- | --- |
| 2-Propanone | 0.785320 | 1160.7 | 1.56 | 945.1 | 1373.6 | 124.9 |
| 2-Butanone | 0.799861 | 1190.8 | 1.31 | 881.7 | 1169.6 | 159.2 |
| 2-Heptanone | 0.811185 | 1262.37 | 1.06 | 773.58 | 968.9 | 242.54 |
| Aniline | 1.017511 | 1637.8 | 0.846 | 366.4 | 467.1 | 194.1 |
| N-methylaniline | 0.982278 | 1563.9 | 0.816 | 416.2 | 520.7 | 207.1 |
| Pyridine | 0.978138 | 1416.2 | 1.024 | 509.7 | 701.9 | 131.5 |

**Table S2: Coefficient of thermal expansion and Isothermal compressibility of Alkanones + Aromatic amines at 298.15K [14,16,22].**

| x_1_ | α _exp._/10^-3^ K^-1^ | α _comp._/10^-3^ K^-1^ | β_T_ exp./ TPa^-1^ | β_T_ comp./ TPa^-1^ |
| --- | --- | --- | --- | --- |
| 2-propanone+ Aniline | | | | |
| 0.0569 | 0.87 | 1.00 | 487.25 | 514.33 |
| 0.1088 | 0.90 | 1.01 | 506.12 | 532.20 |
| 0.1539 | 0.92 | 1.02 | 523.31 | 548.61 |
| 0.1978 | 0.93 | 1.03 | 541.10 | 565.85 |
| 0.2484 | 0.96 | 1.03 | 562.75 | 587.01 |
| 0.3039 | 0.98 | 1.05 | 588.21 | 612.17 |
| 0.3505 | 1.00 | 1.06 | 611.27 | 635.21 |
| 0.4114 | 1.03 | 1.07 | 644.54 | 668.93 |
| 0.4585 | 1.05 | 1.08 | 672.74 | 697.67 |
| 0.5035 | 1.07 | 1.09 | 702.18 | 727.80 |
| 0.5501 | 1.10 | 1.10 | 735.74 | 762.24 |
| 0.5922 | 1.13 | 1.12 | 768.86 | 796.12 |
| 0.6499 | 1.16 | 1.13 | 820.23 | 848.70 |
| 0.6993 | 1.20 | 1.15 | 869.91 | 899.15 |
| 0.7522 | 1.25 | 1.17 | 931.14 | 961.10 |
| 0.8082 | 1.30 | 1.19 | 1005.65 | 1035.60 |
| 0.8533 | 1.35 | 1.21 | 1075.23 | 1104.70 |
| 0.8951 | 1.40 | 1.23 | 1147.72 | 1175.81 |
| 0.9545 | 1.49 | 1.26 | 1267.45 | 1291.92 |
| 2-propanone+N-methylaniline | | | | |
| 0.0440 | 0.84 | 1.03 | 535.21 | 583.60 |
| 0.0984 | 0.86 | 1.04 | 552.88 | 601.28 |
| 0.1447 | 0.87 | 1.05 | 568.87 | 617.46 |
| 0.1996 | 0.89 | 1.06 | 589.11 | 638.11 |
| 0.2503 | 0.91 | 1.07 | 609.35 | 658.87 |
| 0.2919 | 0.93 | 1.07 | 626.86 | 676.68 |
| 0.3511 | 0.95 | 1.08 | 654.65 | 705.16 |
| 0.3924 | 0.97 | 1.09 | 676.06 | 727.08 |
| 0.4520 | 1.01 | 1.10 | 709.72 | 761.16 |
| 0.4948 | 1.03 | 1.11 | 736.40 | 787.92 |
| 0.5563 | 1.07 | 1.13 | 779.52 | 830.88 |
| 0.6004 | 1.10 | 1.14 | 814.31 | 865.23 |
| 0.6473 | 1.14 | 1.15 | 854.87 | 904.70 |
| 0.6962 | 1.18 | 1.17 | 902.59 | 950.92 |
| 0.7506 | 1.23 | 1.18 | 962.73 | 1008.58 |
| 0.8009 | 1.29 | 1.20 | 1026.08 | 1068.83 |
| 0.8518 | 1.35 | 1.22 | 1098.45 | 1136.88 |
| 0.8986 | 1.41 | 1.24 | 1174.16 | 1207.73 |
| 0.9488 | 1.48 | 1.26 | 1266.53 | 1293.85 |
| 2-propanone+pyridine | | | | |
| 0.0514 | 1.05 | 1.09 | 724.85 | 718.54 |
| 0.0969 | 1.07 | 1.10 | 743.25 | 736.39 |
| 0.1486 | 1.09 | 1.10 | 764.55 | 757.84 |
| 0.2023 | 1.11 | 1.11 | 787.18 | 781.24 |
| 0.2444 | 1.13 | 1.12 | 805.61 | 800.61 |
| 0.2960 | 1.14 | 1.13 | 829.29 | 825.69 |
| 0.3544 | 1.17 | 1.14 | 857.84 | 856.01 |
| 0.3965 | 1.19 | 1.14 | 879.50 | 878.83 |
| 0.4516 | 1.21 | 1.16 | 910.00 | 910.82 |
| 0.5036 | 1.23 | 1.17 | 941.02 | 943.08 |
| 0.5448 | 1.26 | 1.17 | 966.96 | 969.72 |
| 0.5940 | 1.28 | 1.18 | 1000.46 | 1004.07 |
| 0.6454 | 1.31 | 1.19 | 1037.72 | 1041.91 |
| 0.6971 | 1.35 | 1.21 | 1078.40 | 1083.29 |
| 0.7518 | 1.38 | 1.22 | 1124.28 | 1129.86 |
| 0.8049 | 1.42 | 1.23 | 1172.01 | 1178.59 |
| 0.8456 | 1.45 | 1.24 | 1210.80 | 1218.61 |
| 0.8955 | 1.48 | 1.26 | 1260.88 | 1271.00 |
| 0.9456 | 1.52 | 1.27 | 1314.08 | 1327.91 |
| 2-butanone+Aniline | | | | |
| 0.0560 | 0.903 | 1.003 | 496.32 | 517.07 |
| 0.1188 | 0.931 | 1.013 | 519.43 | 538.21 |
| 0.1547 | 0.934 | 1.021 | 532.65 | 555.17 |
| 0.2038 | 0.935 | 1.030 | 548.21 | 576.04 |
| 0.2563 | 0.934 | 1.041 | 565.45 | 600.02 |
| 0.3064 | 0.936 | 1.051 | 583.49 | 624.30 |
| 0.3474 | 0.942 | 1.060 | 600.17 | 645.56 |
| 0.4089 | 0.957 | 1.074 | 628.92 | 679.85 |
| 0.4523 | 0.973 | 1.084 | 652.12 | 706.00 |
| 0.5026 | 0.997 | 1.096 | 682.17 | 738.68 |
| 0.5467 | 1.021 | 1.107 | 711.07 | 769.43 |
| 0.6019 | 1.053 | 1.122 | 750.81 | 811.65 |
| 0.6481 | 1.080 | 1.136 | 786.97 | 850.63 |
| 0.7048 | 1.113 | 1.152 | 833.79 | 902.33 |
| 0.7451 | 1.136 | 1.165 | 870.19 | 943.76 |
| 0.8030 | 1.167 | 1.185 | 925.27 | 1008.35 |
| 0.8487 | 1.192 | 1.201 | 972.17 | 1064.42 |
| 0.9019 | 1.223 | 1.221 | 1032.51 | 1136.51 |
| 0.9489 | 1.258 | 1.240 | 1093.58 | 1207.67 |
| 2-butanone+ N-methylaniline | | | | |
| 0.0760 | 0.84 | 1.04 | 544.55 | 596.49 |
| 0.1115 | 0.85 | 1.04 | 556.18 | 609.47 |
| 0.1566 | 0.86 | 1.05 | 571.91 | 626.83 |
| 0.2076 | 0.88 | 1.06 | 590.74 | 647.38 |
| 0.2589 | 0.90 | 1.07 | 611.10 | 669.52 |
| 0.2967 | 0.91 | 1.08 | 627.10 | 686.96 |
| 0.3471 | 0.93 | 1.09 | 649.51 | 711.40 |
| 0.3973 | 0.95 | 1.10 | 673.52 | 737.77 |
| 0.4477 | 0.97 | 1.11 | 699.11 | 766.03 |
| 0.4950 | 0.99 | 1.12 | 725.10 | 795.02 |
| 0.5471 | 1.01 | 1.13 | 755.50 | 829.08 |
| 0.5992 | 1.04 | 1.14 | 788.21 | 865.92 |
| 0.6921 | 1.08 | 1.16 | 853.27 | 939.49 |
| 0.7463 | 1.12 | 1.18 | 896.65 | 988.75 |
| 0.8007 | 1.15 | 1.19 | 944.44 | 1042.78 |
| 0.8470 | 1.18 | 1.21 | 989.17 | 1093.03 |
| 0.8993 | 1.22 | 1.23 | 1044.70 | 1154.79 |
| 0.9472 | 1.26 | 1.24 | 1101.05 | 1216.65 |
| 2-butanone+Pyridine | | | | |
| 0.0517 | 1.041 | 1.089 | 720.25 | 719.67 |
| 0.1009 | 1.051 | 1.097 | 736.14 | 739.63 |
| 0.1510 | 1.061 | 1.104 | 752.94 | 760.79 |
| 0.1952 | 1.071 | 1.111 | 768.23 | 780.06 |
| 0.2473 | 1.083 | 1.120 | 786.89 | 803.63 |
| 0.3010 | 1.095 | 1.128 | 807.09 | 829.18 |
| 0.3559 | 1.109 | 1.137 | 828.61 | 856.42 |
| 0.4041 | 1.121 | 1.146 | 848.35 | 881.43 |
| 0.4494 | 1.132 | 1.154 | 867.52 | 905.73 |
| 0.4994 | 1.146 | 1.162 | 889.53 | 933.65 |
| 0.5472 | 1.159 | 1.171 | 911.43 | 961.44 |
| 0.5916 | 1.171 | 1.179 | 932.63 | 988.35 |
| 0.6951 | 1.203 | 1.198 | 985.19 | 1055.16 |
| 0.7563 | 1.222 | 1.210 | 1018.66 | 1097.79 |
| 0.8029 | 1.238 | 1.220 | 1045.49 | 1132.02 |
| 0.8525 | 1.255 | 1.230 | 1075.20 | 1169.97 |
| 0.8944 | 1.270 | 1.238 | 1101.30 | 1203.33 |
| 0.9525 | 1.292 | 1.251 | 1139.03 | 1251.64 |
| 2-heptanone+Aniline | | | | |
| 0.0619 | 0.869 | 1.009 | 497.79 | 530.78 |
| 0.1115 | 0.882 | 1.022 | 521.13 | 558.43 |
| 0.1601 | 0.891 | 1.034 | 543.46 | 585.95 |
| 0.2133 | 0.899 | 1.048 | 567.37 | 616.23 |
| 0.2575 | 0.905 | 1.058 | 586.91 | 641.37 |
| 0.3064 | 0.911 | 1.070 | 608.83 | 669.79 |
| 0.3638 | 0.920 | 1.083 | 634.67 | 703.27 |
| 0.4002 | 0.925 | 1.091 | 651.04 | 724.31 |
| 0.4537 | 0.935 | 1.103 | 676.17 | 756.42 |
| 0.5092 | 0.946 | 1.115 | 702.89 | 790.16 |
| 0.5430 | 0.954 | 1.122 | 719.64 | 811.11 |
| 0.5964 | 0.967 | 1.134 | 746.85 | 844.88 |
| 0.6599 | 0.984 | 1.147 | 780.14 | 885.88 |
| 0.7057 | 0.997 | 1.157 | 804.78 | 916.17 |
| 0.7584 | 1.012 | 1.168 | 833.76 | 951.98 |
| 0.8041 | 1.024 | 1.178 | 859.19 | 983.73 |
| 0.8515 | 1.036 | 1.188 | 885.79 | 1017.54 |
| 0.9033 | 1.047 | 1.198 | 914.73 | 1055.33 |
| 0.9467 | 1.054 | 1.208 | 938.61 | 1087.63 |
| 2-heptanone+ N-methylaniline | | | | |
| 0.0583 | 0.827 | 1.039 | 542.46 | 597.36 |
| 0.1032 | 0.837 | 1.049 | 559.55 | 618.43 |
| 0.1524 | 0.848 | 1.058 | 578.69 | 641.92 |
| 0.2036 | 0.860 | 1.069 | 599.14 | 666.95 |
| 0.2475 | 0.870 | 1.077 | 616.89 | 688.63 |
| 0.2987 | 0.883 | 1.087 | 638.11 | 714.58 |
| 0.3448 | 0.894 | 1.096 | 657.36 | 738.14 |
| 0.3994 | 0.908 | 1.106 | 680.71 | 766.82 |
| 0.4452 | 0.919 | 1.115 | 700.57 | 791.29 |
| 0.4949 | 0.932 | 1.125 | 722.41 | 818.33 |
| 0.5465 | 0.945 | 1.134 | 745.63 | 847.24 |
| 0.5977 | 0.957 | 1.144 | 768.90 | 876.34 |
| 0.6464 | 0.969 | 1.153 | 791.57 | 904.86 |
| 0.6904 | 0.980 | 1.162 | 812.32 | 931.07 |
| 0.7506 | 0.995 | 1.173 | 841.02 | 967.44 |
| 0.8023 | 1.008 | 1.182 | 866.40 | 999.76 |
| 0.8499 | 1.020 | 1.191 | 890.08 | 1029.97 |
| 0.8989 | 1.033 | 1.200 | 914.95 | 1061.73 |
| 0.9494 | 1.046 | 1.210 | 941.11 | 1095.12 |
| 2-heptanone+ Pyridine | | | | |
| 0.0553 | 1.026 | 1.092 | 719.64 | 728.14 |
| 0.1034 | 1.028 | 1.101 | 734.76 | 752.19 |
| 0.1543 | 1.030 | 1.110 | 750.61 | 777.26 |
| 0.2032 | 1.033 | 1.119 | 765.60 | 801.05 |
| 0.2521 | 1.035 | 1.127 | 780.14 | 824.29 |
| 0.3042 | 1.037 | 1.135 | 795.29 | 848.66 |
| 0.3542 | 1.039 | 1.142 | 809.46 | 871.62 |
| 0.3994 | 1.040 | 1.149 | 821.92 | 891.92 |
| 0.4544 | 1.041 | 1.157 | 836.77 | 916.17 |
| 0.5053 | 1.043 | 1.164 | 850.26 | 938.22 |
| 0.5544 | 1.044 | 1.170 | 862.98 | 959.00 |
| 0.6019 | 1.046 | 1.176 | 875.08 | 978.74 |
| 0.6671 | 1.048 | 1.184 | 891.36 | 1005.21 |
| 0.7047 | 1.049 | 1.188 | 900.64 | 1020.24 |
| 0.7500 | 1.051 | 1.193 | 911.52 | 1037.88 |
| 0.8056 | 1.053 | 1.200 | 924.69 | 1059.17 |
| 0.8528 | 1.055 | 1.205 | 935.63 | 1076.88 |
| 0.8956 | 1.056 | 1.209 | 945.32 | 1092.57 |
| 0.9444 | 1.058 | 1.214 | 956.20 | 1110.22 |

**Table S3: Thermodynamic properties of Alkanone + Aromatic amines at 293.15 K**

| $x_{1}$ | α _comp._/ 10^-3^ K^-1^ | β_T comp_./ TPa^-1^ | $P_{i}$ / MPa | ${\Delta E}_{vap}$/  KJ-mol^-1^ | ${\Delta H}_{vap}$/  KJ-mol^-1^ | CED /  J-mol^-1^ cm^-3^ | δ /  (J-mol^-1^ cm^-3^)^1/2^ | $V_{f}$/  cm^3^-mol^-1^ |
| --- | --- | --- | --- | --- | --- | --- | --- | --- |
| 2-propanone+ Aniline | | | | | | | | |
| 0.0522 | 0.995 | 514.02 | 567.65 | 51.13 | 53.56 | 566.64 | 23.80 | 4.29 |
| 0.1078 | 1.004 | 532.68 | 552.71 | 49.14 | 51.58 | 550.68 | 23.47 | 4.41 |
| 0.1567 | 1.013 | 550.32 | 539.40 | 47.42 | 49.86 | 536.54 | 23.16 | 4.52 |
| 0.2074 | 1.022 | 569.81 | 525.54 | 45.65 | 48.09 | 521.85 | 22.84 | 4.64 |
| 0.2490 | 1.029 | 586.76 | 514.14 | 44.23 | 46.66 | 509.84 | 22.58 | 4.74 |
| 0.3016 | 1.039 | 610.09 | 499.36 | 42.43 | 44.86 | 494.41 | 22.24 | 4.88 |
| 0.3454 | 1.048 | 631.53 | 486.63 | 40.93 | 43.37 | 481.32 | 21.94 | 5.01 |
| 0.3832 | 1.057 | 651.36 | 475.50 | 39.65 | 42.09 | 470.00 | 21.68 | 5.13 |
| 0.4313 | 1.067 | 678.58 | 461.17 | 38.03 | 40.47 | 455.35 | 21.34 | 5.28 |
| 0.4908 | 1.082 | 715.63 | 443.19 | 36.05 | 38.49 | 437.16 | 20.91 | 5.50 |
| 0.5497 | 1.097 | 757.10 | 424.90 | 34.11 | 36.54 | 418.87 | 20.47 | 5.74 |
| 0.5830 | 1.107 | 782.67 | 414.47 | 33.02 | 35.46 | 408.49 | 20.21 | 5.88 |
| 0.6446 | 1.125 | 835.75 | 394.62 | 31.01 | 33.45 | 388.92 | 19.72 | 6.18 |
| 0.6911 | 1.140 | 880.65 | 379.48 | 29.53 | 31.96 | 374.12 | 19.34 | 6.42 |
| 0.7465 | 1.160 | 942.28 | 360.75 | 27.75 | 30.19 | 356.02 | 18.87 | 6.76 |
| 0.8046 | 1.182 | 1015.92 | 341.01 | 25.93 | 28.36 | 337.09 | 18.36 | 7.15 |
| 0.8497 | 1.200 | 1080.69 | 325.60 | 24.54 | 26.98 | 322.42 | 17.96 | 7.49 |
| 0.9045 | 1.225 | 1170.52 | 306.73 | 22.89 | 25.33 | 304.59 | 17.45 | 7.95 |
| 0.9475 | 1.245 | 1250.61 | 291.92 | 21.62 | 24.06 | 290.64 | 17.05 | 8.35 |
| 2-propanone + N-methylaniline | | | | | | | | |
| 0.0565 | 1.030 | 588.24 | 513.18 | 54.69 | 57.12 | 512.64 | 22.64 | 4.75 |
| 0.1032 | 1.036 | 603.24 | 503.60 | 52.79 | 55.23 | 502.60 | 22.42 | 4.84 |
| 0.1494 | 1.043 | 619.05 | 493.95 | 50.93 | 53.37 | 492.54 | 22.19 | 4.93 |
| 0.1916 | 1.050 | 634.59 | 484.87 | 49.24 | 51.67 | 483.10 | 21.98 | 5.03 |
| 0.2510 | 1.059 | 658.14 | 471.83 | 46.88 | 49.32 | 469.65 | 21.67 | 5.17 |
| 0.2952 | 1.067 | 676.98 | 461.97 | 45.16 | 47.59 | 459.52 | 21.44 | 5.28 |
| 0.3555 | 1.078 | 705.06 | 448.14 | 42.82 | 45.25 | 445.33 | 21.10 | 5.44 |
| 0.4039 | 1.087 | 730.16 | 436.56 | 40.95 | 43.39 | 433.61 | 20.82 | 5.58 |
| 0.4478 | 1.097 | 754.84 | 425.84 | 39.28 | 41.71 | 422.80 | 20.56 | 5.72 |
| 0.4981 | 1.108 | 785.38 | 413.39 | 37.38 | 39.82 | 410.24 | 20.25 | 5.90 |
| 0.5489 | 1.119 | 819.52 | 400.44 | 35.49 | 37.93 | 397.30 | 19.93 | 6.09 |
| 0.5965 | 1.131 | 854.42 | 388.14 | 33.75 | 36.19 | 384.99 | 19.62 | 6.28 |
| 0.6485 | 1.145 | 896.82 | 374.33 | 31.87 | 34.31 | 371.31 | 19.27 | 6.51 |
| 0.7018 | 1.160 | 945.16 | 359.92 | 29.98 | 32.42 | 357.08 | 18.90 | 6.77 |
| 0.7472 | 1.175 | 991.67 | 347.22 | 28.39 | 30.82 | 344.67 | 18.57 | 7.02 |
| 0.8020 | 1.193 | 1053.29 | 331.91 | 26.52 | 28.95 | 329.71 | 18.16 | 7.34 |
| 0.8563 | 1.212 | 1122.99 | 316.38 | 24.70 | 27.14 | 314.62 | 17.74 | 7.70 |
| 0.9053 | 1.231 | 1194.72 | 302.07 | 23.10 | 25.54 | 300.81 | 17.34 | 8.07 |
| 0.9548 | 1.252 | 1277.18 | 287.36 | 21.52 | 23.95 | 286.71 | 16.93 | 8.48 |
| 2-propanone + Pyridine | | | | | | | | |
| 0.0577 | 1.082 | 716.55 | 442.61 | 35.42 | 37.86 | 442.41 | 21.03 | 5.51 |
| 0.1007 | 1.088 | 733.14 | 435.10 | 34.67 | 37.11 | 434.75 | 20.85 | 5.60 |
| 0.1536 | 1.096 | 754.42 | 425.90 | 33.77 | 36.21 | 425.37 | 20.62 | 5.72 |
| 0.2043 | 1.104 | 775.76 | 417.11 | 32.91 | 35.35 | 416.42 | 20.41 | 5.84 |
| 0.2219 | 1.107 | 783.45 | 414.05 | 32.61 | 35.05 | 413.31 | 20.33 | 5.89 |
| 0.2968 | 1.119 | 818.16 | 400.85 | 31.35 | 33.78 | 399.92 | 20.00 | 6.08 |
| 0.3537 | 1.128 | 846.77 | 390.68 | 30.39 | 32.83 | 389.69 | 19.74 | 6.24 |
| 0.4051 | 1.138 | 874.01 | 381.54 | 29.54 | 31.97 | 380.50 | 19.51 | 6.39 |
| 0.5191 | 1.159 | 940.69 | 361.15 | 27.66 | 30.10 | 360.04 | 18.97 | 6.75 |
| 0.5519 | 1.165 | 961.64 | 355.25 | 27.13 | 29.56 | 354.14 | 18.82 | 6.86 |
| 0.5969 | 1.174 | 991.87 | 347.12 | 26.40 | 28.83 | 346.05 | 18.60 | 7.02 |
| 0.6425 | 1.184 | 1024.14 | 338.92 | 25.66 | 28.10 | 337.87 | 18.38 | 7.19 |
| 0.7030 | 1.197 | 1070.01 | 328.00 | 24.70 | 27.14 | 327.02 | 18.08 | 7.43 |
| 0.7504 | 1.208 | 1108.44 | 319.46 | 23.96 | 26.39 | 318.54 | 17.85 | 7.63 |
| 0.8066 | 1.221 | 1157.48 | 309.29 | 23.08 | 25.51 | 308.47 | 17.56 | 7.88 |
| 0.8569 | 1.234 | 1205.30 | 300.07 | 22.29 | 24.73 | 299.43 | 17.30 | 8.12 |
| 0.9019 | 1.245 | 1251.04 | 291.83 | 21.60 | 24.04 | 291.35 | 17.07 | 8.35 |
| 0.9456 | 1.257 | 1298.21 | 283.87 | 20.94 | 23.37 | 283.55 | 16.84 | 8.59 |
| 2-butanone + Aniline | | | | | | | | |
| 0.0580 | 0.998 | 518.55 | 563.94 | 51.25 | 53.69 | 562.48 | 23.72 | 4.32 |
| 0.1122 | 1.007 | 539.36 | 547.58 | 49.64 | 52.07 | 545.29 | 23.35 | 4.45 |
| 0.1576 | 1.016 | 556.80 | 534.70 | 48.33 | 50.77 | 531.37 | 23.05 | 4.56 |
| 0.2052 | 1.025 | 576.58 | 520.92 | 46.97 | 49.40 | 516.82 | 22.73 | 4.68 |
| 0.2543 | 1.034 | 598.14 | 506.81 | 45.58 | 48.02 | 502.01 | 22.41 | 4.81 |
| 0.3043 | 1.044 | 621.78 | 492.33 | 44.18 | 46.62 | 487.07 | 22.07 | 4.95 |
| 0.3562 | 1.055 | 648.19 | 477.24 | 42.74 | 45.18 | 471.64 | 21.72 | 5.11 |
| 0.4059 | 1.066 | 675.01 | 462.99 | 41.39 | 43.83 | 457.12 | 21.38 | 5.26 |
| 0.4530 | 1.077 | 702.77 | 449.24 | 40.11 | 42.54 | 443.31 | 21.05 | 5.43 |
| 0.5048 | 1.089 | 735.40 | 434.25 | 38.71 | 41.15 | 428.31 | 20.70 | 5.61 |
| 0.5443 | 1.099 | 762.33 | 422.72 | 37.65 | 40.09 | 416.88 | 20.42 | 5.77 |
| 0.6120 | 1.117 | 812.87 | 402.91 | 35.85 | 38.28 | 397.38 | 19.93 | 6.05 |
| 0.6480 | 1.127 | 842.14 | 392.39 | 34.89 | 37.33 | 387.09 | 19.67 | 6.21 |
| 0.7044 | 1.144 | 892.74 | 375.63 | 33.40 | 35.84 | 370.90 | 19.26 | 6.49 |
| 0.7754 | 1.166 | 962.63 | 355.04 | 31.58 | 34.02 | 351.13 | 18.74 | 6.86 |
| 0.8081 | 1.177 | 1000.43 | 344.96 | 30.70 | 33.13 | 341.53 | 18.48 | 7.07 |
| 0.8493 | 1.192 | 1049.47 | 332.83 | 29.64 | 32.08 | 330.02 | 18.17 | 7.32 |
| 0.9027 | 1.211 | 1119.67 | 317.09 | 28.29 | 30.72 | 315.24 | 17.76 | 7.69 |
| 0.9476 | 1.228 | 1184.29 | 304.06 | 27.17 | 29.60 | 303.01 | 17.41 | 8.02 |
| 2-butanone + N-methylaniline | | | | | | | | |
| 0.0627 | 1.031 | 591.88 | 510.82 | 54.82 | 57.26 | 510.22 | 22.59 | 4.77 |
| 0.1133 | 1.039 | 609.85 | 499.51 | 53.08 | 55.52 | 498.49 | 22.33 | 4.88 |
| 0.1564 | 1.046 | 626.23 | 489.70 | 51.60 | 54.03 | 488.34 | 22.10 | 4.98 |
| 0.2058 | 1.054 | 645.60 | 478.67 | 49.94 | 52.38 | 476.91 | 21.84 | 5.09 |
| 0.2538 | 1.062 | 665.85 | 467.74 | 48.34 | 50.78 | 465.69 | 21.58 | 5.21 |
| 0.3018 | 1.071 | 687.41 | 456.72 | 46.75 | 49.19 | 454.43 | 21.32 | 5.34 |
| 0.3464 | 1.079 | 708.82 | 446.36 | 45.29 | 47.73 | 443.89 | 21.07 | 5.46 |
| 0.3938 | 1.088 | 732.64 | 435.47 | 43.77 | 46.20 | 432.77 | 20.80 | 5.60 |
| 0.4435 | 1.098 | 759.32 | 423.97 | 42.19 | 44.62 | 421.09 | 20.52 | 5.75 |
| 0.4907 | 1.108 | 786.84 | 412.83 | 40.70 | 43.13 | 409.90 | 20.25 | 5.90 |
| 0.5508 | 1.121 | 824.85 | 398.51 | 38.82 | 41.26 | 395.58 | 19.89 | 6.12 |
| 0.5959 | 1.132 | 856.16 | 387.56 | 37.43 | 39.86 | 384.73 | 19.61 | 6.29 |
| 0.6529 | 1.146 | 898.79 | 373.73 | 35.69 | 38.13 | 371.05 | 19.26 | 6.52 |
| 0.7489 | 1.171 | 979.99 | 350.32 | 32.84 | 35.28 | 348.04 | 18.66 | 6.96 |
| 0.7938 | 1.184 | 1022.09 | 339.47 | 31.55 | 33.98 | 337.38 | 18.37 | 7.18 |
| 0.8492 | 1.200 | 1079.44 | 325.89 | 29.97 | 32.41 | 324.18 | 18.00 | 7.48 |
| 0.8981 | 1.215 | 1133.84 | 314.12 | 28.62 | 31.06 | 312.73 | 17.68 | 7.76 |
| 0.9525 | 1.233 | 1202.29 | 300.65 | 27.12 | 27.12 | 299.75 | 17.31 | 8.11 |
| 2-butanone + Pyridine | | | | | | | | |
| 0.0584 | 1.082 | 717.90 | 441.99 | 35.78 | 38.21 | 441.75 | 21.02 | 5.51 |
| 0.1010 | 1.089 | 734.71 | 434.41 | 35.31 | 37.75 | 433.97 | 20.83 | 5.61 |
| 0.1557 | 1.097 | 757.09 | 424.78 | 34.72 | 37.16 | 424.12 | 20.59 | 5.74 |
| 0.2073 | 1.105 | 779.05 | 415.80 | 34.17 | 36.61 | 414.98 | 20.37 | 5.86 |
| 0.2479 | 1.111 | 797.20 | 408.71 | 33.73 | 36.17 | 407.80 | 20.19 | 5.96 |
| 0.2928 | 1.119 | 817.55 | 401.08 | 33.25 | 35.69 | 400.04 | 20.00 | 6.08 |
| 0.3534 | 1.128 | 846.24 | 390.88 | 32.61 | 35.05 | 389.70 | 19.74 | 6.24 |
| 0.3992 | 1.136 | 869.26 | 383.11 | 32.12 | 34.55 | 381.90 | 19.54 | 6.36 |
| 0.4535 | 1.145 | 897.46 | 374.08 | 31.54 | 33.97 | 372.82 | 19.31 | 6.52 |
| 0.4962 | 1.153 | 920.28 | 367.13 | 31.08 | 33.52 | 365.80 | 19.13 | 6.64 |
| 0.5461 | 1.161 | 948.39 | 358.96 | 30.56 | 32.99 | 357.66 | 18.91 | 6.79 |
| 0.5481 | 1.162 | 949.53 | 358.64 | 30.53 | 32.97 | 357.32 | 18.90 | 6.80 |
| 0.6003 | 1.171 | 980.01 | 350.27 | 29.99 | 32.42 | 348.97 | 18.68 | 6.96 |
| 0.6425 | 1.179 | 1005.94 | 343.50 | 29.54 | 31.98 | 342.24 | 18.50 | 7.10 |
| 0.6948 | 1.188 | 1039.29 | 335.23 | 28.99 | 31.43 | 334.05 | 18.28 | 7.27 |
| 0.7267 | 1.194 | 1060.20 | 330.28 | 28.66 | 31.10 | 329.12 | 18.14 | 7.38 |
| 0.7519 | 1.199 | 1077.29 | 326.35 | 28.40 | 30.83 | 325.25 | 18.03 | 7.47 |
| 0.8053 | 1.210 | 1114.67 | 318.14 | 27.84 | 30.28 | 317.13 | 17.81 | 7.66 |
| 0.8550 | 1.220 | 1151.20 | 310.56 | 27.33 | 29.77 | 309.68 | 17.60 | 7.85 |
| 0.8907 | 1.227 | 1178.30 | 305.21 | 26.96 | 29.40 | 304.41 | 17.45 | 7.99 |
| 0.9459 | 1.238 | 1222.71 | 296.88 | 26.39 | 28.83 | 296.30 | 17.21 | 8.21 |
| 2-heptanone + Aniline | | | | | | | | |
| 0.0636 | 1.004 | 532.56 | 552.82 | 52.01 | 54.45 | 551.82 | 23.49 | 4.41 |
| 0.1118 | 1.017 | 558.99 | 533.13 | 51.34 | 53.78 | 531.45 | 23.05 | 4.57 |
| 0.1570 | 1.028 | 584.16 | 515.83 | 50.76 | 53.20 | 513.73 | 22.67 | 4.72 |
| 0.2145 | 1.042 | 616.21 | 495.61 | 50.11 | 52.55 | 493.08 | 22.21 | 4.92 |
| 0.2606 | 1.053 | 642.25 | 480.49 | 49.62 | 52.06 | 477.74 | 21.86 | 5.07 |
| 0.3043 | 1.063 | 666.97 | 467.10 | 49.21 | 51.64 | 464.17 | 21.54 | 5.22 |
| 0.3617 | 1.076 | 700.11 | 450.44 | 48.69 | 51.13 | 447.45 | 21.15 | 5.41 |
| 0.4108 | 1.086 | 728.12 | 437.41 | 48.30 | 50.74 | 434.31 | 20.84 | 5.57 |
| 0.4739 | 1.100 | 765.10 | 421.48 | 47.83 | 50.26 | 418.43 | 20.46 | 5.78 |
| 0.5164 | 1.109 | 790.43 | 411.33 | 47.52 | 49.96 | 408.35 | 20.21 | 5.93 |
| 0.5533 | 1.117 | 812.89 | 402.79 | 47.26 | 49.70 | 399.93 | 20.00 | 6.05 |
| 0.6026 | 1.127 | 843.03 | 391.97 | 46.94 | 49.38 | 389.26 | 19.73 | 6.22 |
| 0.6411 | 1.135 | 867.26 | 383.74 | 46.69 | 49.12 | 381.21 | 19.52 | 6.35 |
| 0.7001 | 1.147 | 904.95 | 371.71 | 46.32 | 48.75 | 369.48 | 19.22 | 6.56 |
| 0.7507 | 1.158 | 938.22 | 361.80 | 46.00 | 48.44 | 359.86 | 18.97 | 6.74 |
| 0.8030 | 1.169 | 973.18 | 352.03 | 45.68 | 48.12 | 350.39 | 18.72 | 6.92 |
| 0.8540 | 1.179 | 1008.52 | 342.75 | 45.38 | 47.82 | 341.52 | 18.48 | 7.11 |
| 0.9011 | 1.189 | 1041.67 | 334.55 | 45.11 | 47.54 | 333.68 | 18.27 | 7.29 |
| 0.9478 | 1.198 | 1075.34 | 326.68 | 44.84 | 47.28 | 326.19 | 18.06 | 7.46 |
| 2-heptanone + N-methylaniline | | | | | | | | |
| 0.0587 | 1.034 | 582.98 | 519.83 | 57.37 | 59.81 | 519.29 | 22.79 | 4.69 |
| 0.1005 | 1.042 | 602.03 | 507.44 | 56.63 | 59.07 | 506.59 | 22.51 | 4.80 |
| 0.1512 | 1.052 | 625.37 | 493.16 | 55.78 | 58.22 | 492.00 | 22.18 | 4.94 |
| 0.2007 | 1.062 | 648.72 | 479.79 | 54.98 | 57.42 | 478.39 | 21.87 | 5.08 |
| 0.2538 | 1.072 | 674.09 | 466.18 | 54.17 | 56.61 | 464.56 | 21.55 | 5.23 |
| 0.3035 | 1.082 | 698.43 | 453.94 | 53.43 | 55.87 | 452.18 | 21.26 | 5.37 |
| 0.3507 | 1.090 | 721.8 | 442.87 | 52.76 | 55.20 | 441.00 | 21.00 | 5.50 |
| 0.3960 | 1.099 | 744.88 | 432.54 | 52.13 | 54.57 | 430.62 | 20.75 | 5.63 |
| 0.4512 | 1.109 | 773.11 | 420.64 | 51.41 | 53.85 | 418.66 | 20.46 | 5.79 |
| 0.4897 | 1.117 | 793.54 | 412.49 | 50.91 | 53.34 | 410.53 | 20.26 | 5.91 |
| 0.5627 | 1.130 | 832.79 | 397.82 | 50.00 | 52.44 | 395.92 | 19.90 | 6.13 |
| 0.6097 | 1.139 | 858.52 | 388.84 | 49.45 | 51.88 | 386.99 | 19.67 | 6.27 |
| 0.6603 | 1.148 | 887.03 | 379.43 | 48.86 | 51.29 | 377.70 | 19.43 | 6.42 |
| 0.7049 | 1.156 | 912.48 | 371.46 | 48.36 | 50.79 | 369.83 | 19.23 | 6.56 |
| 0.7497 | 1.164 | 938.75 | 363.64 | 47.86 | 50.30 | 362.15 | 19.03 | 6.70 |
| 0.7986 | 1.173 | 967.77 | 355.43 | 47.34 | 49.78 | 354.09 | 18.82 | 6.86 |
| 0.8512 | 1.183 | 999.95 | 346.81 | 46.79 | 49.22 | 345.70 | 18.59 | 7.03 |
| 0.9019 | 1.192 | 1031.3 | 338.88 | 46.28 | 48.72 | 337.96 | 18.38 | 7.19 |
| 0.9468 | 1.200 | 1059.7 | 332.05 | 45.84 | 48.27 | 331.32 | 18.20 | 7.34 |
| 2-heptanone + Pyridine | | | | | | | | |
| 0.0575 | 1.085 | 707.26 | 449.69 | 37.71 | 40.15 | 449.56 | 21.20 | 5.42 |
| 0.1074 | 1.094 | 731.39 | 438.52 | 38.07 | 40.50 | 438.29 | 20.94 | 5.56 |
| 0.1529 | 1.102 | 753.00 | 429.04 | 38.40 | 40.84 | 428.73 | 20.71 | 5.68 |
| 0.2040 | 1.111 | 777.01 | 419.06 | 38.77 | 41.21 | 418.69 | 20.46 | 5.82 |
| 0.2522 | 1.119 | 799.17 | 410.31 | 39.13 | 41.57 | 409.89 | 20.25 | 5.94 |
| 0.3071 | 1.127 | 823.86 | 401.05 | 39.55 | 41.99 | 400.56 | 20.01 | 6.08 |
| 0.3542 | 1.134 | 844.47 | 393.69 | 39.93 | 42.36 | 393.18 | 19.83 | 6.19 |
| 0.4587 | 1.149 | 889.67 | 378.59 | 40.74 | 43.18 | 378.02 | 19.44 | 6.44 |
| 0.5105 | 1.156 | 911.06 | 371.90 | 41.16 | 43.60 | 371.31 | 19.27 | 6.55 |
| 0.5455 | 1.160 | 925.42 | 367.56 | 41.45 | 43.89 | 367.00 | 19.16 | 6.63 |
| 0.6023 | 1.167 | 948.45 | 360.85 | 41.91 | 44.35 | 360.26 | 18.98 | 6.75 |
| 0.6542 | 1.174 | 968.90 | 355.12 | 42.34 | 44.77 | 354.53 | 18.83 | 6.86 |
| 0.6999 | 1.179 | 986.43 | 350.37 | 42.72 | 45.16 | 349.79 | 18.70 | 6.96 |
| 0.7551 | 1.185 | 1007.30 | 344.91 | 43.19 | 45.63 | 344.34 | 18.56 | 7.07 |
| 0.8207 | 1.192 | 1031.50 | 338.82 | 43.75 | 46.19 | 338.26 | 18.39 | 7.19 |
| 0.8551 | 1.196 | 1043.85 | 335.81 | 44.05 | 46.48 | 335.26 | 18.31 | 7.26 |
| 0.8984 | 1.200 | 1059.27 | 332.14 | 44.42 | 46.86 | 331.60 | 18.21 | 7.34 |
| 0.9585 | 1.206 | 1079.81 | 327.39 | 44.96 | 47.39 | 326.86 | 18.08 | 7.44 |

**Table S4: Thermodynamic properties of Alkanone + Aromatic amines at 303.15 K**

| $x_{1}$ | α _comp._/ 10^-3^ K^-1^ | β_T comp_. / TPa^-1^ | $P_{i}$ / MPa | ${\Delta E}_{vap}$/  KJ-mol^-1^ | ${\Delta H}_{vap}$/  KJ-mol^-1^ | CED /  J-mol^-1^ cm^-3^ | δ /  (J-mol^-1^ cm^-3^)^1/2^ | $V_{f}$ /  cm^3^-mol^-1^ |
| --- | --- | --- | --- | --- | --- | --- | --- | --- |
| 2-propanone+ Aniline | | | | | | | | |
| 0.0515 | 1.006 | 523.03 | 583.17 | 52.99 | 55.51 | 582.10 | 24.13 | 4.32 |
| 0.1002 | 1.014 | 540.17 | 569.23 | 51.16 | 53.68 | 567.20 | 23.82 | 4.43 |
| 0.1475 | 1.023 | 557.91 | 555.60 | 49.39 | 51.91 | 552.63 | 23.51 | 4.54 |
| 0.1926 | 1.031 | 576.27 | 542.27 | 47.71 | 50.23 | 538.60 | 23.21 | 4.65 |
| 0.2399 | 1.040 | 596.54 | 528.39 | 45.98 | 48.50 | 523.94 | 22.89 | 4.77 |
| 0.3024 | 1.052 | 626.11 | 509.56 | 43.72 | 46.24 | 504.32 | 22.46 | 4.95 |
| 0.3475 | 1.062 | 650.22 | 495.32 | 42.07 | 44.59 | 489.80 | 22.13 | 5.09 |
| 0.3947 | 1.073 | 676.11 | 481.02 | 40.41 | 42.93 | 474.96 | 21.79 | 5.24 |
| 0.4503 | 1.086 | 710.18 | 463.61 | 38.45 | 40.97 | 457.16 | 21.38 | 5.44 |
| 0.4979 | 1.099 | 743.37 | 448.00 | 36.77 | 39.29 | 441.50 | 21.01 | 5.63 |
| 0.5502 | 1.113 | 783.22 | 430.79 | 34.95 | 37.47 | 424.29 | 20.60 | 5.85 |
| 0.6037 | 1.129 | 829.06 | 412.79 | 33.11 | 35.63 | 406.44 | 20.16 | 6.11 |
| 0.6464 | 1.143 | 870.51 | 397.96 | 31.63 | 34.15 | 391.92 | 19.80 | 6.33 |
| 0.6975 | 1.160 | 925.31 | 380.15 | 29.90 | 32.42 | 374.54 | 19.35 | 6.63 |
| 0.7548 | 1.181 | 994.66 | 360.09 | 27.99 | 30.51 | 355.09 | 18.84 | 7.00 |
| 0.8087 | 1.203 | 1070.12 | 340.87 | 26.22 | 28.74 | 336.66 | 18.35 | 7.39 |
| 0.8567 | 1.225 | 1148.92 | 323.18 | 24.66 | 27.18 | 319.96 | 17.89 | 7.80 |
| 0.9014 | 1.246 | 1230.77 | 306.92 | 23.24 | 25.76 | 304.63 | 17.45 | 8.21 |
| 0.9494 | 1.271 | 1330.93 | 289.43 | 21.75 | 24.27 | 288.25 | 16.98 | 8.71 |
| 2-propanone + N-methylaniline | | | | | | | | |
| 0.0461 | 1.040 | 596.42 | 528.47 | 56.99 | 59.51 | 527.96 | 22.98 | 4.77 |
| 0.0934 | 1.047 | 612.33 | 518.13 | 54.97 | 57.49 | 517.15 | 22.74 | 4.86 |
| 0.1524 | 1.056 | 633.75 | 504.95 | 52.46 | 54.98 | 503.36 | 22.44 | 4.99 |
| 0.1937 | 1.062 | 649.93 | 495.49 | 50.73 | 53.25 | 493.52 | 22.22 | 5.09 |
| 0.2442 | 1.071 | 671.80 | 483.34 | 48.60 | 51.12 | 481.09 | 21.93 | 5.21 |
| 0.2834 | 1.078 | 688.71 | 474.41 | 47.01 | 49.53 | 471.77 | 21.72 | 5.31 |
| 0.3357 | 1.088 | 714.52 | 461.50 | 44.85 | 47.37 | 458.54 | 21.41 | 5.46 |
| 0.3991 | 1.101 | 748.81 | 445.55 | 42.28 | 44.80 | 442.31 | 21.03 | 5.66 |
| 0.4460 | 1.110 | 776.08 | 433.76 | 40.43 | 42.95 | 430.34 | 20.74 | 5.81 |
| 0.4904 | 1.121 | 805.94 | 421.64 | 38.64 | 41.16 | 418.19 | 20.45 | 5.98 |
| 0.5507 | 1.136 | 849.15 | 405.45 | 36.29 | 38.81 | 401.92 | 20.05 | 6.22 |
| 0.5949 | 1.147 | 884.75 | 393.15 | 34.58 | 37.11 | 389.70 | 19.74 | 6.41 |
| 0.6467 | 1.162 | 930.92 | 378.43 | 32.61 | 35.13 | 375.10 | 19.37 | 6.66 |
| 0.6928 | 1.176 | 976.36 | 365.14 | 30.89 | 33.41 | 362.01 | 19.03 | 6.90 |
| 0.7424 | 1.192 | 1031.18 | 350.48 | 29.06 | 31.58 | 347.67 | 18.65 | 7.19 |
| 0.7993 | 1.212 | 1102.18 | 333.41 | 27.01 | 29.53 | 331.02 | 18.19 | 7.56 |
| 0.8469 | 1.230 | 1169.52 | 318.90 | 25.34 | 27.86 | 316.97 | 17.80 | 7.90 |
| 0.8977 | 1.251 | 1250.39 | 303.30 | 23.60 | 26.12 | 301.91 | 17.38 | 8.31 |
| 0.9484 | 1.274 | 1343.47 | 287.40 | 21.90 | 24.42 | 286.70 | 16.93 | 8.77 |
| 2-propanone + Pyridine | | | | | | | | |
| 0.0493 | 1.096 | 736.64 | 451.07 | 36.50 | 39.02 | 450.86 | 21.23 | 5.59 |
| 0.0954 | 1.103 | 755.63 | 442.54 | 35.66 | 38.18 | 442.15 | 21.03 | 5.70 |
| 0.1463 | 1.111 | 777.71 | 433.08 | 34.74 | 37.26 | 432.53 | 20.80 | 5.82 |
| 0.1983 | 1.119 | 801.46 | 423.42 | 33.80 | 36.32 | 422.68 | 20.56 | 5.95 |
| 0.2424 | 1.127 | 822.77 | 415.16 | 33.01 | 35.53 | 414.32 | 20.35 | 6.07 |
| 0.2929 | 1.135 | 848.34 | 405.74 | 32.11 | 34.63 | 404.78 | 20.12 | 6.21 |
| 0.3522 | 1.146 | 880.58 | 394.54 | 31.06 | 33.58 | 393.49 | 19.84 | 6.39 |
| 0.4048 | 1.156 | 910.86 | 384.67 | 30.13 | 32.65 | 383.51 | 19.58 | 6.55 |
| 0.4497 | 1.164 | 938.49 | 376.14 | 29.35 | 31.87 | 374.96 | 19.36 | 6.70 |
| 0.4970 | 1.174 | 969.15 | 367.18 | 28.53 | 31.05 | 366.00 | 19.13 | 6.86 |
| 0.5412 | 1.183 | 999.27 | 358.84 | 27.77 | 30.29 | 357.64 | 18.91 | 7.02 |
| 0.5965 | 1.195 | 1039.72 | 348.32 | 26.83 | 29.35 | 347.12 | 18.63 | 7.24 |
| 0.6594 | 1.209 | 1089.24 | 336.37 | 25.77 | 28.29 | 335.25 | 18.31 | 7.49 |
| 0.6912 | 1.216 | 1116.23 | 330.25 | 25.23 | 27.75 | 329.20 | 18.14 | 7.63 |
| 0.7491 | 1.230 | 1168.49 | 319.11 | 24.27 | 26.79 | 318.21 | 17.84 | 7.90 |
| 0.8475 | 1.255 | 1266.95 | 300.32 | 22.66 | 25.18 | 299.71 | 17.31 | 8.39 |
| 0.8946 | 1.268 | 1319.68 | 291.28 | 21.90 | 24.42 | 290.88 | 17.06 | 8.65 |
| 0.9443 | 1.282 | 1379.55 | 281.74 | 21.10 | 23.62 | 281.54 | 16.78 | 8.95 |
| 2-butanone + Aniline | | | | | | | | |
| 0.0582 | 1.008 | 527.73 | 579.27 | 53.10 | 55.62 | 578.11 | 24.04 | 4.35 |
| 0.1097 | 1.018 | 548.47 | 562.76 | 51.46 | 53.98 | 560.66 | 23.68 | 4.48 |
| 0.1485 | 1.026 | 564.73 | 550.56 | 50.25 | 52.77 | 547.74 | 23.40 | 4.58 |
| 0.2050 | 1.037 | 589.50 | 533.11 | 48.52 | 51.04 | 529.30 | 23.01 | 4.73 |
| 0.2507 | 1.046 | 610.14 | 519.53 | 47.17 | 49.69 | 514.88 | 22.69 | 4.85 |
| 0.3064 | 1.057 | 637.74 | 502.57 | 45.51 | 48.03 | 497.10 | 22.30 | 5.01 |
| 0.3426 | 1.065 | 657.11 | 491.42 | 44.44 | 46.96 | 485.60 | 22.04 | 5.13 |
| 0.4140 | 1.081 | 698.23 | 469.55 | 42.36 | 44.88 | 463.28 | 21.52 | 5.37 |
| 0.4470 | 1.089 | 718.74 | 459.46 | 41.41 | 43.93 | 453.07 | 21.29 | 5.49 |
| 0.4910 | 1.100 | 748.38 | 445.74 | 40.13 | 42.65 | 439.34 | 20.96 | 5.65 |
| 0.5537 | 1.117 | 793.97 | 426.40 | 38.34 | 40.86 | 420.10 | 20.50 | 5.91 |
| 0.6044 | 1.131 | 834.47 | 410.78 | 36.91 | 39.43 | 404.65 | 20.12 | 6.14 |
| 0.6434 | 1.142 | 868.73 | 398.57 | 35.80 | 38.32 | 392.74 | 19.82 | 6.32 |
| 0.7038 | 1.161 | 927.02 | 379.62 | 34.10 | 36.63 | 374.40 | 19.35 | 6.64 |
| 0.7716 | 1.183 | 1000.41 | 358.54 | 32.23 | 34.75 | 354.10 | 18.82 | 7.03 |
| 0.8079 | 1.196 | 1044.38 | 347.15 | 31.23 | 33.75 | 343.25 | 18.53 | 7.26 |
| 0.8418 | 1.211 | 1096.50 | 334.70 | 30.19 | 32.71 | 332.00 | 18.22 | 7.53 |
| 0.8988 | 1.230 | 1169.06 | 318.99 | 28.77 | 31.29 | 316.64 | 17.79 | 7.90 |
| 0.9466 | 1.250 | 1244.49 | 304.38 | 27.51 | 30.03 | 302.91 | 17.40 | 8.28 |
| 2-butanone + N-methylaniline | | | | | | | | |
| 0.0641 | 1.043 | 603.76 | 523.65 | 56.65 | 59.17 | 522.97 | 22.87 | 4.81 |
| 0.1123 | 1.051 | 621.77 | 512.22 | 54.90 | 57.42 | 511.08 | 22.61 | 4.92 |
| 0.1581 | 1.058 | 639.80 | 501.36 | 53.26 | 55.78 | 499.78 | 22.36 | 5.03 |
| 0.2036 | 1.066 | 658.89 | 490.42 | 51.64 | 54.16 | 488.50 | 22.10 | 5.14 |
| 0.2591 | 1.076 | 683.71 | 477.01 | 49.68 | 52.20 | 474.70 | 21.79 | 5.28 |
| 0.3061 | 1.085 | 706.31 | 465.51 | 48.04 | 50.56 | 462.92 | 21.52 | 5.41 |
| 0.3513 | 1.093 | 729.49 | 454.37 | 46.48 | 49.00 | 451.59 | 21.25 | 5.55 |
| 0.4022 | 1.104 | 757.02 | 441.92 | 44.76 | 47.28 | 438.90 | 20.95 | 5.70 |
| 0.4472 | 1.113 | 783.88 | 430.52 | 43.23 | 45.75 | 427.44 | 20.67 | 5.85 |
| 0.5025 | 1.125 | 818.78 | 416.67 | 41.39 | 43.91 | 413.51 | 20.34 | 6.05 |
| 0.5536 | 1.137 | 853.98 | 403.73 | 39.71 | 42.23 | 400.56 | 20.01 | 6.24 |
| 0.5984 | 1.148 | 887.04 | 392.38 | 38.26 | 40.78 | 389.25 | 19.73 | 6.42 |
| 0.6489 | 1.161 | 927.27 | 379.55 | 36.65 | 39.17 | 376.53 | 19.40 | 6.64 |
| 0.6969 | 1.174 | 969.47 | 367.09 | 35.13 | 37.65 | 364.26 | 19.09 | 6.87 |
| 0.7543 | 1.190 | 1024.72 | 352.14 | 33.34 | 35.86 | 349.61 | 18.70 | 7.16 |
| 0.8047 | 1.205 | 1077.80 | 339.05 | 31.81 | 34.33 | 336.87 | 18.35 | 7.43 |
| 0.8497 | 1.219 | 1128.85 | 327.48 | 30.46 | 32.98 | 325.57 | 18.04 | 7.70 |
| 0.8965 | 1.235 | 1187.56 | 315.26 | 29.08 | 31.60 | 313.77 | 17.71 | 7.99 |
| 0.9524 | 1.254 | 1264.35 | 300.78 | 27.47 | 29.99 | 299.79 | 17.31 | 8.38 |
| 2-butanone + Pyridine | | | | | | | | |
| 0.0589 | 1.098 | 741.01 | 449.07 | 36.73 | 39.25 | 448.83 | 21.19 | 5.61 |
| 0.1170 | 1.107 | 765.68 | 438.17 | 36.06 | 38.58 | 437.68 | 20.92 | 5.75 |
| 0.1620 | 1.114 | 785.52 | 429.85 | 35.54 | 38.06 | 429.18 | 20.72 | 5.86 |
| 0.2056 | 1.121 | 805.45 | 421.84 | 35.04 | 37.56 | 421.03 | 20.52 | 5.97 |
| 0.2489 | 1.128 | 826.16 | 413.88 | 34.54 | 37.06 | 412.99 | 20.32 | 6.09 |
| 0.3075 | 1.138 | 855.14 | 403.32 | 33.87 | 36.39 | 402.28 | 20.06 | 6.25 |
| 0.3506 | 1.145 | 877.29 | 395.66 | 33.38 | 35.90 | 394.56 | 19.86 | 6.37 |
| 0.4016 | 1.154 | 904.40 | 386.72 | 32.80 | 35.32 | 385.54 | 19.64 | 6.52 |
| 0.4494 | 1.162 | 931.49 | 378.26 | 32.25 | 34.77 | 377.06 | 19.42 | 6.66 |
| 0.4959 | 1.171 | 958.61 | 370.20 | 31.73 | 34.25 | 369.01 | 19.21 | 6.81 |
| 0.5482 | 1.180 | 990.29 | 361.28 | 31.14 | 33.66 | 360.12 | 18.98 | 6.98 |
| 0.5967 | 1.189 | 1020.95 | 353.11 | 30.60 | 33.12 | 351.96 | 18.76 | 7.14 |
| 0.6484 | 1.199 | 1055.36 | 344.44 | 30.02 | 32.54 | 343.36 | 18.53 | 7.32 |
| 0.6972 | 1.209 | 1089.38 | 336.34 | 29.47 | 31.99 | 335.37 | 18.31 | 7.49 |
| 0.7446 | 1.218 | 1123.58 | 328.63 | 28.95 | 31.47 | 327.73 | 18.10 | 7.67 |
| 0.7952 | 1.228 | 1162.21 | 320.40 | 28.39 | 30.91 | 319.67 | 17.88 | 7.87 |
| 0.8476 | 1.239 | 1203.77 | 312.07 | 27.81 | 30.34 | 311.46 | 17.65 | 8.08 |
| 0.8928 | 1.249 | 1241.29 | 304.97 | 27.32 | 29.84 | 304.51 | 17.45 | 8.26 |
| 0.9371 | 1.258 | 1279.72 | 298.07 | 26.84 | 29.36 | 297.76 | 17.26 | 8.46 |
| 2-heptanone + Aniline | | | | | | | | |
| 0.0610 | 1.014 | 540.46 | 569.01 | 53.93 | 56.45 | 568.05 | 23.83 | 4.43 |
| 0.1087 | 1.027 | 567.66 | 548.43 | 53.20 | 55.72 | 546.76 | 23.38 | 4.60 |
| 0.1563 | 1.039 | 595.14 | 529.32 | 52.54 | 55.06 | 527.14 | 22.96 | 4.76 |
| 0.2061 | 1.052 | 624.24 | 510.70 | 51.90 | 54.42 | 508.14 | 22.54 | 4.94 |
| 0.2539 | 1.063 | 651.41 | 494.64 | 51.39 | 53.91 | 491.75 | 22.18 | 5.10 |
| 0.3051 | 1.075 | 682.76 | 477.51 | 50.79 | 53.31 | 474.44 | 21.78 | 5.28 |
| 0.3592 | 1.088 | 715.5 | 461.02 | 50.24 | 52.76 | 457.86 | 21.40 | 5.47 |
| 0.4079 | 1.099 | 744.73 | 447.38 | 49.80 | 52.32 | 444.09 | 21.07 | 5.63 |
| 0.4483 | 1.108 | 769.69 | 436.45 | 49.45 | 51.97 | 433.18 | 20.81 | 5.77 |
| 0.4949 | 1.123 | 810.56 | 419.84 | 48.83 | 51.35 | 419.28 | 20.48 | 6.00 |
| 0.5420 | 1.129 | 829.13 | 412.76 | 48.67 | 51.19 | 409.70 | 20.24 | 6.11 |
| 0.5872 | 1.139 | 858.87 | 402.00 | 48.31 | 50.83 | 399.12 | 19.98 | 6.27 |
| 0.6512 | 1.153 | 901.35 | 387.70 | 47.83 | 50.35 | 385.09 | 19.62 | 6.50 |
| 0.7078 | 1.165 | 940.29 | 375.60 | 47.42 | 49.94 | 373.31 | 19.32 | 6.71 |
| 0.7546 | 1.175 | 973.13 | 366.05 | 47.08 | 49.61 | 364.05 | 19.08 | 6.89 |
| 0.8054 | 1.186 | 1009.9 | 356.01 | 46.73 | 49.25 | 354.37 | 18.82 | 7.08 |
| 0.8543 | 1.196 | 1046.2 | 346.69 | 46.38 | 48.91 | 345.43 | 18.59 | 7.27 |
| 0.9018 | 1.207 | 1082.1 | 338.03 | 46.07 | 48.59 | 337.14 | 18.36 | 7.46 |
| 0.9474 | 1.216 | 1117.5 | 329.97 | 45.76 | 48.28 | 329.46 | 18.15 | 7.64 |
| 2-heptanone + N-methylaniline | | | | | | | | |
| 0.0580 | 1.045 | 608.62 | 520.50 | 57.91 | 60.43 | 519.95 | 22.80 | 4.84 |
| 0.1036 | 1.054 | 630.65 | 506.80 | 57.09 | 59.61 | 505.91 | 22.49 | 4.97 |
| 0.1519 | 1.064 | 654.34 | 492.98 | 56.25 | 58.77 | 491.77 | 22.18 | 5.11 |
| 0.2010 | 1.074 | 679.03 | 479.47 | 55.43 | 57.95 | 478.02 | 21.86 | 5.26 |
| 0.2137 | 1.076 | 685.35 | 476.15 | 55.23 | 57.75 | 474.62 | 21.79 | 5.29 |
| 0.2927 | 1.092 | 726.17 | 455.93 | 53.99 | 56.51 | 454.13 | 21.31 | 5.53 |
| 0.3480 | 1.103 | 755.55 | 442.57 | 53.17 | 55.69 | 440.64 | 20.99 | 5.69 |
| 0.4002 | 1.113 | 783.84 | 430.53 | 52.42 | 54.94 | 428.53 | 20.70 | 5.85 |
| 0.4486 | 1.123 | 810.47 | 419.87 | 51.76 | 54.28 | 417.82 | 20.44 | 6.00 |
| 0.4960 | 1.132 | 837.22 | 409.77 | 51.12 | 53.64 | 407.72 | 20.19 | 6.15 |
| 0.5469 | 1.142 | 866.85 | 399.22 | 50.45 | 52.98 | 397.23 | 19.93 | 6.31 |
| 0.5959 | 1.151 | 895.77 | 389.51 | 49.84 | 52.36 | 387.59 | 19.69 | 6.47 |
| 0.6416 | 1.160 | 923.46 | 380.72 | 49.27 | 51.79 | 378.88 | 19.46 | 6.62 |
| 0.6955 | 1.170 | 956.66 | 370.76 | 48.63 | 51.15 | 369.07 | 19.21 | 6.80 |
| 0.7531 | 1.181 | 993.13 | 360.50 | 47.96 | 50.48 | 358.98 | 18.95 | 6.99 |
| 0.7965 | 1.189 | 1021.22 | 353.04 | 47.48 | 50.00 | 351.67 | 18.75 | 7.14 |
| 0.8458 | 1.199 | 1053.76 | 344.83 | 46.93 | 49.45 | 343.65 | 18.54 | 7.31 |
| 0.8996 | 1.209 | 1090.13 | 336.16 | 46.36 | 48.88 | 335.21 | 18.31 | 7.50 |
| 0.9456 | 1.218 | 1121.76 | 329.03 | 45.88 | 48.40 | 328.27 | 18.12 | 7.66 |
| 2-heptanone + Pyridine | | | | | | | | |
| 0.0552 | 1.100 | 746.03 | 446.80 | 37.79 | 40.31 | 446.67 | 21.13 | 5.64 |
| 0.1049 | 1.109 | 771.50 | 435.69 | 38.14 | 40.66 | 435.43 | 20.87 | 5.78 |
| 0.1534 | 1.118 | 796.19 | 425.52 | 38.49 | 41.01 | 425.19 | 20.62 | 5.92 |
| 0.2024 | 1.126 | 820.67 | 415.96 | 38.84 | 41.36 | 415.56 | 20.39 | 6.06 |
| 0.2505 | 1.134 | 844.31 | 407.19 | 39.20 | 41.72 | 406.75 | 20.17 | 6.19 |
| 0.3015 | 1.142 | 869.01 | 398.48 | 39.58 | 42.10 | 398.00 | 19.95 | 6.33 |
| 0.3503 | 1.150 | 892.06 | 390.73 | 39.95 | 42.47 | 390.21 | 19.75 | 6.45 |
| 0.3953 | 1.156 | 912.98 | 383.99 | 40.30 | 42.82 | 383.45 | 19.58 | 6.56 |
| 0.5114 | 1.173 | 965.25 | 368.29 | 41.21 | 43.73 | 367.70 | 19.18 | 6.84 |
| 0.5608 | 1.179 | 986.68 | 362.27 | 41.61 | 44.13 | 361.68 | 19.02 | 6.96 |
| 0.6034 | 1.185 | 1005.00 | 357.31 | 41.96 | 44.48 | 356.72 | 18.89 | 7.05 |
| 0.6656 | 1.192 | 1031.07 | 350.51 | 42.47 | 44.99 | 349.92 | 18.71 | 7.19 |
| 0.7036 | 1.197 | 1046.57 | 346.61 | 42.79 | 45.31 | 346.02 | 18.60 | 7.27 |
| 0.7463 | 1.202 | 1063.90 | 342.36 | 43.14 | 45.66 | 341.79 | 18.49 | 7.36 |
| 0.8019 | 1.208 | 1085.79 | 337.17 | 43.62 | 46.14 | 336.60 | 18.35 | 7.48 |
| 0.8558 | 1.213 | 1106.78 | 332.36 | 44.07 | 46.59 | 331.82 | 18.22 | 7.58 |
| 0.8996 | 1.218 | 1123.33 | 328.68 | 44.45 | 46.97 | 328.15 | 18.11 | 7.67 |
| 0.9516 | 1.223 | 1142.56 | 324.52 | 44.90 | 47.42 | 324.00 | 18.00 | 7.77 |


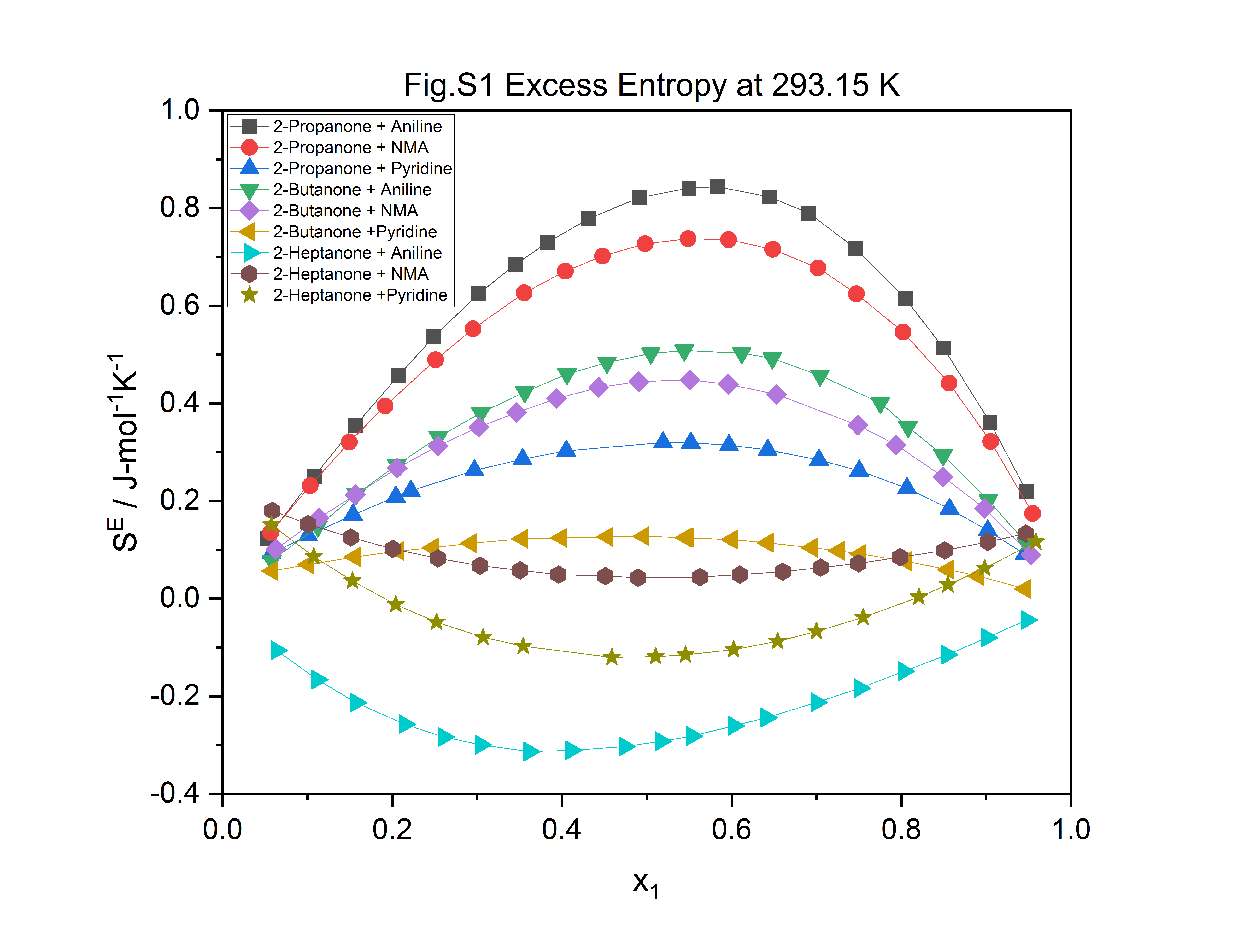


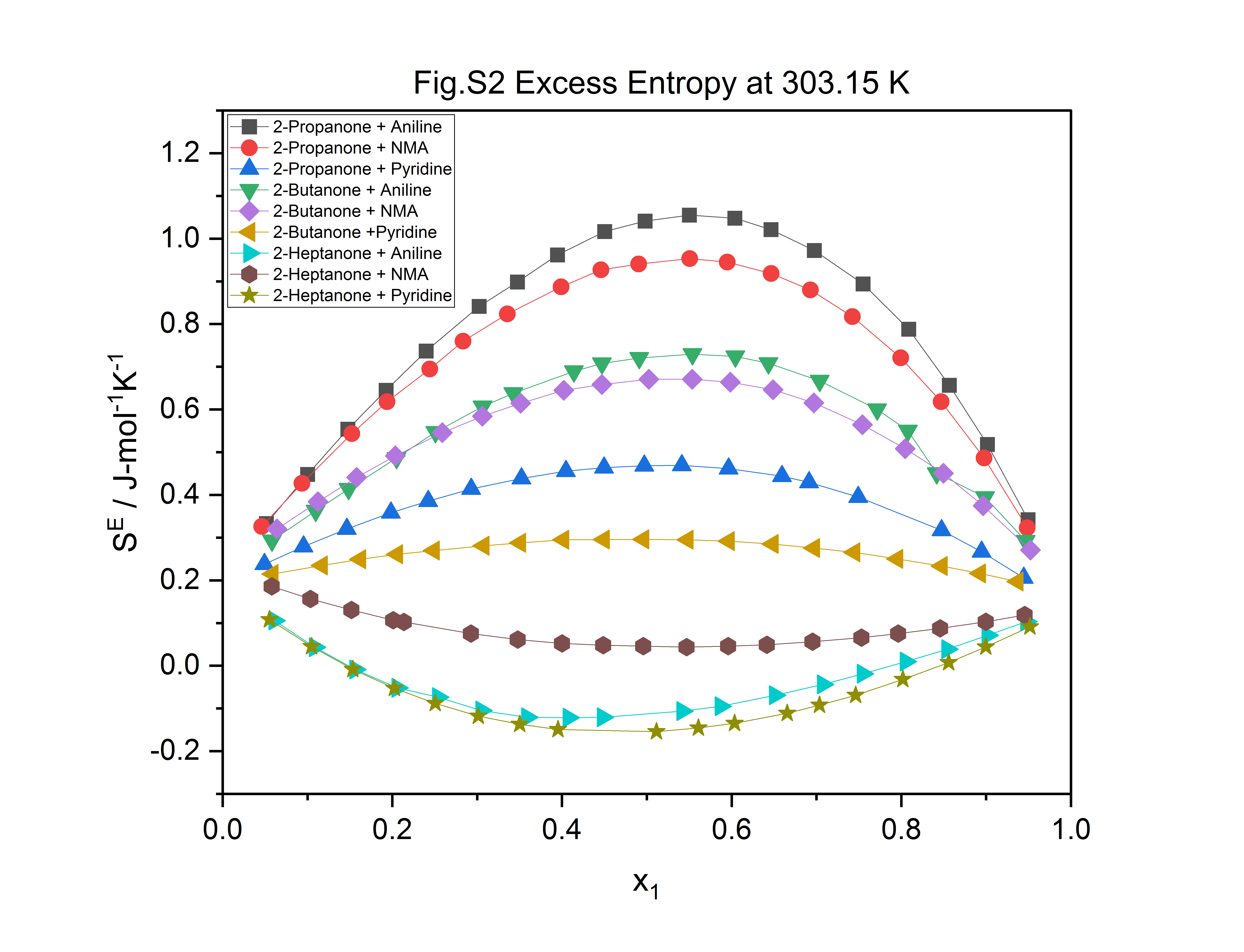

Supplement: Supplementary file 1 [file DataSheet1.docx]
